# Integrative LC-HR-QTOF-MS and Computational Metabolomics Approaches for Compound Annotation, Chemometric Profiling and In Silico Antibacterial Evaluation of Ugandan Propolis

**DOI:** 10.3390/metabo16020109

**Published:** 2026-02-03

**Authors:** Ivan Kahwa, Christina Seel, Ronnie Tumwesigye, Patrick Onen, Ramona Oehme, Susan Billig, Rapheal Wangalwa, Jonans Tusiimire, Claudia Wiesner, Leonard Kaysser

**Affiliations:** 1 Institute for Drug Discovery, Department of Pharmaceutical Biology, Faculty of Medicine, Leipzig University, 04317 Leipzig, Germany; christina.seel@uni-leipzig.de (C.S.); leonard.kaysser@uni-leipzig.de (L.K.); 2 Department of Pharmacy, Faculty of Medicine, Mbarara University of Science and Technology, Mbarara P.O. Box 1410, Uganda; jonanstusiimire@must.ac.ug; 3 Institute of Analytical Chemistry, Faculty of Chemistry, Leipzig University, 04103 Leipzig, Germany; roehme@uni-leipzig.de (R.O.); billig@uni-leipzig.de (S.B.); birkemeyer@chemie.uni-leipzig.de (C.W.); 4 Department of Chemistry, College of Natural Sciences, Makerere University, Kampala P.O. Box 7062, Uganda; ronnie.tumwesigye@mak.ac.ug; 5 Department of Chemistry, Faculty of Science, Kyambogo University, Kyambogo P.O. Box 1, Uganda; ponen@kyu.ac.ug; 6 Department of Biology, Faculty of Science, Mbarara University of Science and Technology, Mbarara P.O. Box 1410, Uganda; wangarapha@must.ac.ug; 7 German Center for Integrative Biodiversity Research (iDiv) Halle-Leipzig-Jena, 04103 Leipzig, Germany

**Keywords:** Ugandan propolis, untargeted metabolomics, molecular networking, chemometric analysis, flavonoids, diterpenoids, in silico antibacterial activity

## Abstract

**Background/Objectives**: Propolis is a complex bee product with a composition that varies according to local vegetation, environmental conditions, and bee foraging behaviours. Recently, gas chromatography–mass spectrometry (GC–MS) has been employed in Uganda to analyse its volatile components. This study examined Ugandan propolis non-volatile metabolites to determine chemotypes and identify antibacterial compounds. **Methods:** Ethanolic extracts were analysed using liquid chromatography–high-resolution quadrupole time-of-flight mass spectrometry (LC-HR-QTOF-MS) in an untargeted MS/MS mode. Data processing was carried out using MZmine, then annotated with Global Natural Products Social Molecular Networking (GNPS) and SIRIUS. Chemometric methods assisted in identifying regional chemical signatures. Metabolites highlighted by the heatmap were evaluated for antibacterial activity using molecular docking against bacterial targets, followed by ADMET (absorption, distribution, metabolism, excretion, and toxicity) assessments. **Results:** Out of 3252 features, 234 and 52 putative compounds were annotated in GNPS and SIRIUS, respectively, as indicated by molecular networking, suggesting high chemical complexity. The chemical space mainly comprises flavonoids (including glycosides, aglycones, methylated, and prenylated derivatives), phenolic acids, amides, hydroxycinnamate derivatives, lignans, megastigmanes, and various diterpenoid skeletons. Multivariate analyses clearly distinguish geographical chemotypes, separating flavonoid-rich regions from diterpenoid-rich regions. Docking studies revealed flavonoids, diterpenoids, and lignans with strong predicted antibacterial activities and favourable ADMET profiles. **Conclusions:** This study provides the first LC–MS characterisation of the non-volatile metabolome of Ugandan propolis, thereby expanding its chemical diversity. Metabolomics and computational approaches lay a foundation for future ecological, chemotaxonomic, and pharmacological research.

## 1. Introduction

Propolis, called “bee glue,” is a sticky substance honeybees gather from plant secretions, buds, and excretions, then mix with wax and saliva to seal and protect hives [1]. Alongside its role as a protective barrier within the hive against microbial invasion, pests, and environmental stressors, propolis has attracted considerable scientific interest due to its varied therapeutic potential [2,3]. Literature has demonstrated a number of studies on its biological activities, including antimicrobial, antioxidant, anti-inflammatory, immunomodulatory, anticancer, and wound-healing effects [4]. These pharmacological properties stem from the plant’s complex and variable phytochemical composition, influenced by ecological and biological factors [5,6]. The local flora, climate, seasonal variations, and the foraging behaviour of bees affect the chemical quality of propolis [7,8]. As a result, propolis shows distinct geographical patterns, and various types have been categorised worldwide. In temperate regions, for example, the extensively studied poplar-type propolis is abundant in flavonoid aglycones and phenolic acids [9]. On the other hand, tropical propolis often contains unique prenylated phenylpropanoids, terpenes, and lignans originating from indigenous plant species [10]. In contrast to the extensive research conducted on European, Brazilian, and Asian propolis, African propolis continues to be insufficiently studied [11].

Uganda is distinguished by its remarkable botanical and ecological diversity, encompassing a broad spectrum of agroecological zones, including wet farmlands, highland forests, savannah rangelands, and semi-arid regions [12,13]. These ecological gradients likely provide honeybees with a variety of resin sources; however, systematic studies are deficient regarding the phytochemical composition of Ugandan propolis [14,15]. Gathering high-quality chemical data from these underutilised sources is essential, not only for evaluating the value of propolis as a natural resource but also for linking various phytochemical patterns to potential biomedical applications and regional standardisation efforts [1,16]. Traditionally, propolis has been examined using gas chromatography–mass spectrometry (GC-MS), a method highly effective for analysing volatile and semi-volatile constituents, such as mono- and sesquiterpenes, simple phenolics, and hydrocarbons [17]. GC-MS cannot reliably detect non-volatile and thermally unstable compounds, limiting its effectiveness for comprehensive propolis analysis [18]. Recently, we used a dual GC-MS workflow to identify compounds in Ugandan propolis, including hydrocarbons, terpenes, phenolics, sugars, and fatty acids. We recommend adding techniques such as LC-MS/MS to broaden the analysis [19].

Many polar, thermolabile, or high-molecular-weight constituents, such as flavonoid glycosides, phenolic acid esters, and prenylated phenolics, are either underrepresented or completely absent from GC-MS analyses [20]. This analytical bias limits our understanding of the non-volatile components of propolis, which are increasingly recognised as vital to its pharmacological effects. Advances in liquid chromatography coupled with high-resolution mass spectrometry (LC-HRMS), particularly utilising quadrupole time-of-flight (QTOF) analysers, currently offer substantial opportunities for comprehensive metabolomics of bee products [21]. LC-HR-QTOF-MS offers high mass accuracy, isotopic fidelity, and comprehensive MS/MS spectra, enabling confident identification of complex metabolites. It excels at detecting non-volatile, polar metabolites above 1000 u, unlike GC-MS. Using gentle electrospray ionisation and specific chromatographic techniques, such as reversed-phase and hydrophilic interaction liquid chromatography, it preserves delicate functional groups and separates phenolic glycosides, flavonoid conjugates, caffeoylquinic acids, and other polar phenolics [22]. Indeed, recent studies on propolis from Asia and South America have emphasised the predominance of polar compounds in LC-HRMS profiles, thereby highlighting the need to move beyond analyses that focus solely on volatile constituents [23,24].

However, interpreting complex samples like propolis involves more than acquiring high-resolution data; it also requires systematic compound annotation. Untargeted metabolomics often yields thousands of spectral features, most of which remain unannotated due to no reference standards [25]. Integrated computational metabolomics workflows have been developed to address these bottlenecks, combining molecular formula prediction, in silico fragmentation, spectral library searches, and molecular networking [26]. Tools such as SIRIUS/CSI: FingerID, MS-FINDER, and MS-DIAL now support annotation at various confidence levels by using accurate masses, isotopic patterns and fragmentation trees to predict tentative compounds at Metabolite Standard Initiative level 3 (MSI level 3) [27]. Community platforms such as Global Natural Products Social Molecular Networking (GNPS) and MassBank provide public spectral databases for the dereplication of MSI level 2 putatively assigned matches [27]. These methods are vital in natural product research, where structural novelty and the avoidance of redundant discoveries are key. For example, a study on Australian propolis employed computational metabolomics to delineate chemical diversity and propose structures in the absence of authentic standards [28].

Metabolomics also relies on chemometric methods, which are essential for analysing complex propolis metabolomes. Techniques such as PCA, PLS-DA, hierarchical clustering, and OPLS-DA help identify patterns, categorise samples by origin, and pinpoint marker metabolites that distinguish groups [19]. Beyond chemical profiling, computational screening for bioactivity complements it. As antibiotic resistance rises, interest in natural antimicrobials increases. Propolis has compounds that may inhibit bacteria, but testing each is slow. Molecular docking and virtual screening evaluate molecular binding using 3D structures and estimate binding energies quickly. For instance, a study docked “neoflavonoid-1” to bacterial proteins, with scores of −7.1 to −7.2 kcal/mol, and showed larger inhibition zones against Gram-positive bacteria in vitro, indicating docking can predict activity and guide experiments [29].

Modern in silico pipelines now go beyond docking energies, incorporating ADMET and toxicity predictions to identify safe, effective leads, as confirmed by a neoflavonoid-1, which showed no predicted acute toxicity, consistent with its in vitro bioactivity [29]. Prenylated phenylpropanoids from Brazilian green propolis, like artepillin C derivatives, were docked to oral *Streptococcus* protein targets. The best ligands formed stable hydrogen bonds, with scores comparable to those of known ligands. All leads met Lipinski’s rules and had good ADMET profiles, indicating oral bioavailability. These cases show how in silico antibacterial tests can prioritise propolis compounds with high target affinity and drug-like properties, speeding up antimicrobial development [30].

The integration of in silico screening with metabolomics is widely used; a case in point is the LC-HRMS analysis of Turkish *Salvia* extracts, which identified rosmarinic acid, which was then subjected to docking studies against bacterial folate enzymes, such as dihydrofolate reductase and dihydropteroate synthase, demonstrating strong binding affinity and thereby corroborating its potential antibacterial activity [31]. These findings show that LC-HRMS metabolite annotation, with targeted docking, links natural products to biological functions. For propolis, using chemometrically identified metabolites for docking against pathogens helps identify compounds responsible for antimicrobial effects. We expand our workflow with LC-HR-QTOF-MS and computational metabolomics to include in silico antibacterial testing of Ugandan propolis. Our goal is to develop detailed chemical profiles, especially of polar compounds not accessible by GC methods. We combine chemometric analysis, molecular docking, and ADMET predictions to connect metabolites with antibacterial activity. This integration aims to characterise chemical diversity, ecological influences on African propolis, and identify promising compounds for antimicrobial drugs.

## 2. Materials and Methods

### 2.1. Chemicals and Propolis Sample Collection from Different Districts of Uganda

The chemicals used were both HPLC-grade (ethanol) and LC-MS grade (acetonitrile, acetic acid, and purified water), purchased from VWR International, Darmstadt, Germany. We used raw propolis samples from nine districts across Uganda, including Nakasongola (NAK), Masindi (MAS), Bushenyi (BUS), Kibuku (KIB), Mbarara (MBA), Lira (LIR), Adjumani (ADJ), Rwampara (RWA), and Kotido (KOT), each representing different ecological zones, as previously described by Kahwa et al. [19]. In each district, samples were collected from three sites managed by experienced beekeepers. These locations differ in geography, climate, altitude, vegetation, rainfall, temperature, soils, and size, all of which significantly affect the quality and quantity of bee propolis. A summary of these factors is available in the Supplementary Table S1. The samples were sorted to remove foreign materials such as honeycomb, dried plant matter, and dried insects, then packed in black polythene bags, stored in a cool, dry place, and kept away from direct sunlight.

### 2.2. Ultrasound-Assisted Extraction and Development of an HPLC-DAD Gradient for Propolis Samples

A 100 mg sample of powdered propolis was combined with 1 mL of 70% ethanol and sonicated for 30 min at 25 °C in an ultrasonic bath. The mixture was then vortexed for 15 min and centrifuged at 13,200 rpm for 15 min. The supernatant was carefully filtered through 0.45 μm membrane filters and centrifuged again for 5 min at 13,200 rpm. A 400-μL aliquot of this extract was transferred into HPLC vials with inserts for analysis. To develop an effective reversed-phase gradient for LC-MS/MS, we initially used an HPLC Hitachi Primade system with a 1430 diode array detector (Dalian, China) (coarse slit width, 400 ms sampling) and a 1210 autosampler (syringe speed = 3, syringe volume = 0.1 mL, wash port speed = 4, fast needle descent, wash port stroke = 3, needle wash time = 1 s), coupled with an 1110 pump (pressure limit 10–300 bar) and a 1310 column oven. Chromatography was performed on a Nucleodur 100–5 C_18_ ec column (250 mm length, 4 mm ID, 5 µm particles, 110 Å pore size, Macherey-Nagel, Düren, Germany). The mobile phase included 0.2% acetic acid (solvent A) and 100% acetonitrile (solvent B), following this gradient: 0–3 min, 25–30% B; 3–5 min, 30% B; 5–12 min, 30–35% B; 12–15 min, 35–40% B; 15–19 min, 40–45% B; 19–25 min, 45–85% B; 25–35 min, 85–90% B; 35–40 min, 90–95% B; and 40–90 min, 95% B. The flow rate was set at 1 mL/min. Diode-array detection was performed over 190–450 nm, with monitoring at 290 nm and an integration range of 250–380 nm for 90 min. The column temperature was maintained at 30 °C, and the injection volume was 20 μL. Data were processed using Primade software (Version 2.0). This optimised gradient, adjusted after multiple trials, was used with modifications for LC-MS/MS analysis.

### 2.3. LC-HR-QTOF Analysis and MS^2^ Data Acquisition for Propolis Extracts

The analyses were performed on a Dionex UltiMate 3000 UHPLC (Thermo Scientific, Waltham, MA, USA) coupled to an electrospray (ESI) High-Resolution Q-Time-of-Flight (HR-QTOF) Impact II (Bruker Daltonics Inc., Bremen, Germany). Bruker Hystar 3.2 software and Bruker Compass 1.8 were used to control the LC-HRMS system. We maintained the same column parameters as outlined above (Section 2.2), except for the gradient, flow rate, and injection volume, which were adjusted to match the instrument parameters. Therefore, in our analysis, acetonitrile (A) and 0.2% acetic acid (B) were used as mobile phases with a gradient of 0 min at 75% B; 22 min at 55% B; 32 min at 15% B; 52 min at 0% B; 60 min at 0% B; 61 min at 75% B; and 70 min at 75% B. The flow rate was set to 0.6 mL/min. The samples were cooled to 10 °C in the autosampler, and the injection volume was 10 µL. MS- and Auto MSMS mass spectra were acquired at the *m*/*z* range of 50–1500 using an electrospray ionisation interface in positive and negative mode. The MS source parameters were 8.5 L/min dry gas flow, the nebulizer gas pressure was set to 3.5 bar, and the dry heater temperature was 250 °C. The capillary voltage of the ion source was set to 3700 V (positive mode) and 2500 V (negative mode). The source transfer time was varied between 52.5 and 82.5 μs, and the prepulse storage time was set to 5 μs. Collison RF was ramped between 200 and 800 Vpp, and the quadrupole MS collision energy was varied by stepping from 80% to 120% during the analysis. In the Auto MSMS mode, the precursor ion mass range was *m*/*z* 50–125, and 3 precursor ions were examined; they were excluded after 3 spectra. The repetition rate of the spectra was set to 1 Hz. Internal instrument calibration was performed utilising ESI-L Tuning Mix (G1969-85000, Agilent Technologies, Santa Clara, CA, USA). All our analyses were performed using three extraction replicates.

### 2.4. Data Pre-Processing Workflow Using MZmine

Raw positive mode UHPLC–QTOF MS/MS data (.d format, Bruker Daltonics) were processed using MZmine version 4.7.29 with the built-in Processing Wizard (mzwizard) [32]. The wizard established a standardised, reproducible LC–MS/MS workflow with parameters optimised for the UHPLC–QTOF system. In the wizard interface, the sample introduction was configured as UHPLC with electrospray ionisation (ESI, positive mode) under data-dependent acquisition (DDA). The chromatographic and data collection settings included enabled smoothing, consistent ionisation across samples, a retention time range from 0.3 to 60.0 min, up to 15 peaks per chromatogram, at least four consecutive scans, a feature FWHM of approximately 0.05 min, an intra-sample RT tolerance of 0.04 min, and a sample-to-sample RT tolerance of 0.10 min. For the QTOF MS setup, data were processed in positive ion mode with absolute intensity scaling. Noise thresholds were set at 5.0 × 10^2^ for MS^1^ and 1.0 × 10^2^ for MS^2^, with a minimum feature height of 1.0 × 10^3^. Mass accuracy tolerances included a scan-to-scan *m*/*z* tolerance of 0.0050 Da (20 ppm), an intra-sample *m*/*z* tolerance of 0.0015 Da (3 ppm), and a sample-to-sample *m*/*z* tolerance of 0.0040 Da (8 ppm) to account for inter-run mass drift while maintaining high alignment specificity. Under the Filters tab, the original feature list was eliminated, allowing a minimum aligned sample threshold of one (0.0%) to retain all valid features. In the annotation section, no local or spectral library files were uploaded. The DDA workflow was selected, with all recommended modules executed, including spectral networking (FBMN/IIMN), exporting for molecular networking and library matching (GNPS) and exporting for structure annotation with SIRIUS (Version 6.3) [33]. The wizard then automatically configured all downstream modules, including mass detection, ADAP chromatogram creation, peak deconvolution, isotope and adduct grouping, feature alignment, gap filling, and feature list export [32].

### 2.5. GNPS Compound Annotation and Molecular Networking

Compound annotation and spectral clustering were performed using Feature-Based Molecular Networking (FBMN) on the Global Natural Products Social Molecular Networking (GNPS) platform (https://gnps.ucsd.edu/ProteoSAFe/) accessed on 30 September 2025, following a workflow previously developed by Wang et al. [33]. The .mgf and .csv files were uploaded together to the FBMN workflow on the GNPS platform, using WinSCP (version 6.3.3 by Martin Přikryl) as the FTP client [34]. The parameters were configured as follows: precursor ion mass tolerance of 2.0 Da, fragment ion mass tolerance of 0.5 Da, a minimum cosine score of 0.7, at least 6 matched fragment ions, a maximum shift of 1999 Da, network TopK set to 10, a minimum cluster size of 2, and MSCluster was enabled to merge highly similar MS^2^ spectra. To prevent over-connected clusters, the maximum size of connected components was limited to 100. In library spectral matching, the parameters were configured to require at least 6 matched peaks and a score threshold of 0.7. The analogue search feature was turned off (Search Analogues = Do Not Search), and the maximum allowed mass difference for analogue searches was set to 100 Da. These settings were designed to ensure high confidence matches to reference spectra and to reduce false analogue annotations. The molecular networks were visualised in the GNPS environment and then exported to Cytoscape v3.10.1 for further curation and visualisation. Nodes representing MS^2^ spectra were linked based on cosine similarity and shared fragment ion patterns, allowing the clustering of structurally related metabolites. Nodes matched to libraries were annotated using GNPS spectral libraries, which helped dereplicate known compounds and identify potential metabolites in Ugandan propolis extracts.

### 2.6. Compound Annotation and Structure Elucidation Using SIRIUS

In silico compound annotation and structural prediction were carried out using SIRIUS v6.3.2 (https://bio.informatik.uni-jena.de/software/sirius/), accessed on 5 October 2025, which includes built-in CSI: FingerID, CANOPUS, and MSNovelist, to achieve high-confidence metabolite identification and classification. The MS^2^ spectral data (.mgf) from MZmine were imported into SIRIUS to calculate molecular formulas, analyse fragmentation patterns, and predict structures. In the Global Configuration, the instrument was configured as QTOF with a mass accuracy of 10 ppm for MS^2^. The fallback adducts included [M + H]^+^, [M + Na]^+^, and [M + K]^+^. Search databases used were BioCyc, ChEBI, COCONUT, FoodDB, KEGG, KNApSAcK, LOTUS, LipidMaps, Maconda, MeSH, MiMeDB, NORMAN, PlantCyc, PubChem (Bio and Metabolites), SuperNatural, and TeroMoL. For the Spectral Library Search, the precursor deviation was set to 20 ppm, and the analogue search was enabled to include structurally related spectra along with exact matches. In the Molecular Formula Identification module, a de novo bottom-up strategy was used, with predictions limited to *m*/*z* values below 400. The elemental composition filter allowed H, C, N, O, and P for de novo results, and the autodetect feature was activated to include additional elements, such as B, Mg, S, Cl, Fe, Zn, Se, and Br, when they appeared.

For compound property prediction, CSI: FingerID was employed to identify fingerprint-based substructures, whereas CANOPUS was used to predict compound classes, with a score threshold filter to retain only high-confidence results. In structure database searching, PubChem served as the fallback database, and the confidence mode was configured to ‘Approximate’. Moreover, MSNovelist was used to generate de novo molecular structures, facilitating deep-learning–assisted reconstruction of novel scaffolds absent from spectral databases. Confidence thresholds were set as follows: a ZODIAC score between 0.6 and 0.7, a CANOPUS class prediction of at least 0.8, a spectral match cosine of 0.8 or higher, and a CSI: FingerID match probability between 0.6 and 0.7. Only compounds meeting these criteria were classified as high-confidence or putatively identified metabolites. This integrated annotation pipeline enabled a comprehensive identification of molecular formulas, substructures, compound classes, and de novo predicted structures, thereby improving the reliability and interpretability of metabolomic profiling of Ugandan propolis extracts.

### 2.7. Chemometric Profiling of Annotated Metabolites

A chemometric analysis explored relationships among annotated metabolites and variability in Ugandan propolis extract compositions. Putative compounds identified via GNPS and SIRIUS were cross-referenced against their peak areas in the Mzmine feature list, thereby combining qualitative and quantitative data. Feature matching used *m*/*z*, RTs, and feature IDs. Only features detected in at least three extraction replicates were included in the analysis. Two csv files were generated: one for GNPS-annotated compounds with the fields Name, RT, *m*/*z*, Class, Adduct, SMILES, InChI, Shared Peaks, GNPS Link; and another for SIRIUS-annotated compounds with the fields Name, Formula, Mass, RT, Class, SMILES, InChI, InChI Key, StructurePerIDRank, FormulaRank, Confidencescore, CSI: FingerIDScore, ZodiacScore, Siriusscore, and Adduct.

The quantitative datasets were imported into MetaboAnalyst 6.0 (https://www.metaboanalyst.ca) on 15 October 2025 for multivariate analysis. The data were normalised by sum, followed by a square-root transformation to mitigate heteroscedasticity. Subsequently, the data were auto-scaled, mean-centred, and divided by each variable’s standard deviation to ensure equal importance of all features. Supervised Partial Least Squares Discriminant Analysis (sPLS-DA) and Orthogonal Partial Least Squares Discriminant Analysis (oPLS-DA) were conducted to identify metabolites that differentiate between propolis samples. Model accuracy and stability were evaluated using cross-validation and permutation tests. VIP scores and correlation loadings were computed to pinpoint the key compounds driving sample clustering. Heatmap visualisation was performed utilising Euclidean distance and Ward’s linkage algorithm to discern sample grouping patterns and metabolite correlations. We constructed a debiased sparse partial-correlation (DSPC) network utilising data from both annotations. The data were normalised by summing, cube-root-transformed, and auto-scaled [35]. This approach eliminates indirect, confounded, or spurious connections often found in high-dimensional omics data, allowing for more precise identification of true biochemical relationships among metabolites [35].

### 2.8. Selection of Chemical Markers from Heatmap Analysis for In Silico Antibacterial Screening

To identify the best candidates, we used a workflow shown in Supplementary Figure S1 to select compounds from GNPS and SIRIUS for potential bioactivity investigation. A literature review was conducted to determine whether these metabolites had been previously reported to exhibit antibacterial activity, using PubMed, Google Scholar, and Scopus with name, InChI Key, SMILES, and keywords such as “antibacterial,” “antimicrobial,” or “bactericidal.” Compounds known to have antibacterial effects were excluded from further assessment. Annotated compounds that were shortlisted but lacked documented antibacterial activity were further evaluated. They were analysed with heatmap analysis in MetaboAnalyst 6.0 (https://www.metaboanalyst.ca), accessed on 16 November 2025, to explore their role in the metabolic diversity of propolis samples. The data preprocessing followed the same normalisation steps as before, including sum normalisation, square-root transformation, and auto-scaling. The heatmap displayed the distribution patterns of compounds without reported antibacterial activity across sites. Compounds with high abundance (red on the heatmap) and found in at least two locations were potential marker metabolites, possibly explaining compositional differences. These were selected for further in silico testing as candidate antibacterial compounds in Ugandan propolis.

### 2.9. Computational Validation of Selected Putative Chemical Markers Through Molecular Docking

To further evaluate the antibacterial activity of the proposed chemical markers, in silico molecular docking was conducted on specific bacterial target proteins essential for microbial survival and pathogenicity. The study included protein targets from both Gram-negative bacteria (such as *Escherichia coli*, *Klebsiella pneumoniae*, and *Pseudomonas aeruginosa*) and Gram-positive bacteria (including *Staphylococcus aureus*, *Bacillus subtilis*, and *Streptococcus mutans*). Seven (07) ligands for this analysis were selected from markers identified in Section 2.8, based on a heatmap of compounds with limited or no antibacterial activity. These markers belonged to the flavonoids, diterpenoids, and lignans chemical classes.

#### 2.9.1. Ligands and Targets Preparation

For our in silico studies involving the selected ligands and targets, as described in Section 2.9, we used MzDOCK (V2.4) for ligand preparation, protein setup, molecular docking, and initial visualisation [36]. Protein targets were sourced from the Protein Data Bank (http://www.rcsb.org/), accessed on 25 October 2025. The targets included *S. aureus* (1JII), *B. subtilis* (1BOW), *S. mutans* (3AIC), *E. coli* (1G27), *K. pneumoniae* (6T77), and *P. aeruginosa* (1U1Z), with their structure files downloaded in (.pdb) format. These were chosen based on their reported resistance profiles in the literature. The subsequent steps in protein preparation comprised the addition of hydrogen atoms and charges, the removal of water molecules, and the elimination of co-crystallised ligands or unnecessary heteroatoms. The grid box was configured based on the co-crystallised ligand and was supported by a review of relevant literature. For proteins without ligands, Prankweb.cz (https://prankweb.cz/), accessed on 25 October 2025 was utilised to predict the binding pocket. The chemical marker compounds, as detailed in Section 2.9, were obtained in (.SDF) format from PubChem. To verify the procedure, the co-crystallised ligands were redocked to identify the scoring function that yielded the lowest Root Mean Square Deviation (RMSD). Additionally, two well-known antibiotics, Ciprofloxacin and Vancomycin, served as positive controls since they target the organisms. After confirming the algorithm’s validity, molecular docking was carried out, and the results were examined using the software’s integrated tools. Extended 3D visualisations were generated with ChimeraX (Version 1.0) (https://www.cgl.ucsf.edu/chimerax/, accessed on 30 September 2025) [37].

#### 2.9.2. ADME and Toxicity Investigation on the Selected Chemical Biomarkers

This was mainly conducted to evaluate the pharmacokinetic properties of the ligands by calculating Absorption, Distribution, Metabolism, and Excretion (ADME) parameters and drug-likeness metrics for all compounds using ADMETLAB 3.0. Additionally, toxicity profiles were predicted using the ProTox-II web server (https://tox.charite.de/protox3/, accessed on 30 September 2025), a computational platform for detailed toxicological assessment accessed on 25 October 2025. Briefly, SMILES-format chemical structures were uploaded to the server to predict various toxicological and molecular endpoints, including hepatotoxicity, neurotoxicity, nephrotoxicity, respiratory toxicity, cardiotoxicity, carcinogenicity, immunotoxicity, mutagenicity, cytotoxicity, and endocrine-related targets like the androgen receptor, aromatase, oestrogen receptor, and peroxisome proliferator-activated receptor gamma (PPAR-γ). Predictions were generated using a combined approach that included molecular similarity analysis, pharmacophore-based modelling, and machine learning algorithms trained on publicly available toxicological data. The study also evaluated stress-response and signalling endpoints, including Nrf2/ARE, HSE, and mitochondrial membrane potential (MMP). Each endpoint received a probability score, and the predicted acute oral toxicity (LD_50_) for each compound was reported.

## 3. Results

### 3.1. HPLC-DAD Chromatogram Developed from the Gradient

**Figure 1:**
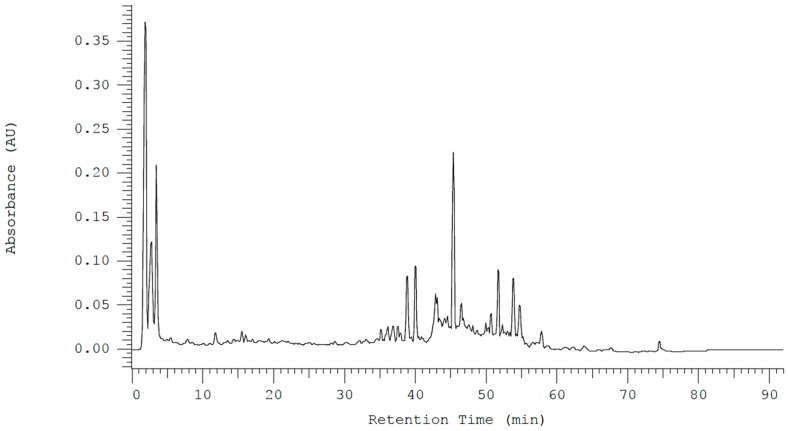
HPLC-DAD representative chromatogram of 70% ethanolic propolis extract at 290 nm shows an early polar peak (2–3 min), phenolic compounds (35–45 min), and flavonoids around 40–55 min during a 90 min gradient on the C_18_ column.

To enable efficient chemical profiling of Ugandan propolis, we optimised the HPLC-DAD gradient and produced a well-resolved chromatogram for the 70% ethanol extract (Figure 1). The chromatogram revealed a highly complex mixture typical of resin-rich bee products, with strong early peaks at 2–5 min corresponding to very polar compounds extracted during the initial isocratic phase. The baseline remained relatively stable until about 35–60 min, after which several medium-intensity peaks appeared, indicating the elution of moderately polar compounds such as phenolic acids, chalcones, and less polar flavonoid derivatives as the acetonitrile concentration increased. After 60 min, only minor peaks were observed, consistent with the elution of highly hydrophobic components during the high-organic phase.

### 3.2. LC–MS Chromatographic Overview

**Figure 2:**
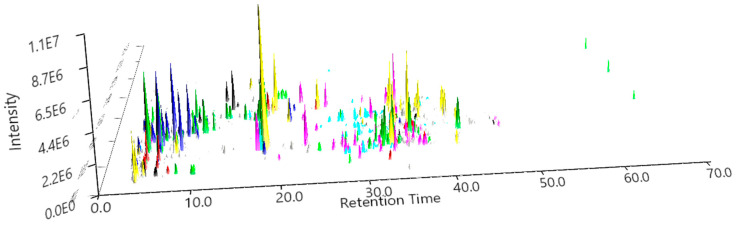
LC–MS chromatogram (MZmine) of propolis extracts from nine Ugandan districts, illustrating distinct ion-intensity patterns over the retention time. Each district is represented by a different colour: ADJ (green), LIR (dark blue), BUS (magenta), KIB (lime), MAS (black), MBA (yellow), NAK (olive), RWA (maroon), and KOT (cyan), emphasising noticeable chemical differences between regions.

The 3D LC–MS/MS chromatogram generated in MZmine revealed significant chemical differences among propolis samples from nine districts in Uganda (Figure 2). All samples showed a dense cluster of early-eluting, high-intensity ions (0–10 min), indicating abundant low- to medium-polarity compounds common to all regions. In the subsequent elution phase, each district exhibited distinct patterns. MBA (yellow) and LIR (dark blue) showed some of the highest peaks between 15 and 25 min, while KIB (lime) and ADJ (green) demonstrated strong signals lasting up to about 50 min, indicating the presence of more mid- to late-eluting semi-polar compounds. Three samples, namely RWA (maroon), NAK (olive), and MAS (black), demonstrated lower overall intensities but exhibited distinct peak clusters within the 20–35 min range, indicating site-specific chemical signatures. In contrast, BUS (magenta) and KOT (cyan) displayed intermediate intensities, with numerous characteristic peaks within the 20–40 min range. The three-dimensional chromatogram demonstrates clear chemical variability among regions, with each district exhibiting unique peak patterns and intensity profiles throughout the chromatographic space.

### 3.3. MS/MS Precursor-Ion Distribution

**Figure 3:**
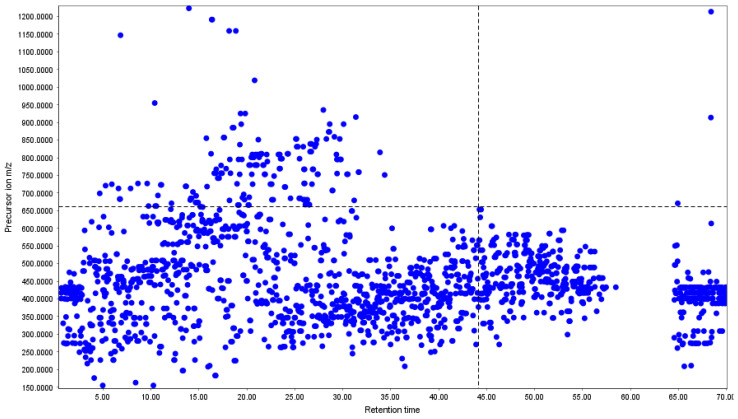
MS/MS precursor-ion scatter plot of all samples showing the distribution of fragmentation events across retention time (x-axis) and precursor *m*/*z* (y-axis). Most precursor ions occur between 5 and 45 min and *m*/*z* 200–500, with fewer events above *m*/*z* 750, reflecting the chemical diversity of the propolis extract.

The MS/MS precursor-ion scatter plot for all samples from the batch provides an overview of fragmentation events during the LC–MS/MS run (Figure 3). Precursor ions are distributed across the retention time, with most MS/MS-triggered features occurring between 5 and 45 min, reflecting the mid-polarity profile typical of propolis components. A smaller, secondary band between *m*/*z* 500–750 may correspond to the presence of heavier flavonoids, prenylated derivatives, and some diterpenoids. Regarding the retention behaviour, early precursor ions eluting before 10 min were mainly low-mass species. In contrast, compounds eluting between 20 and 45 min included both medium- and high-mass ions, indicating increased hydrophobicity as the chromatographic gradient progressed. A small group of late-eluting precursors, around 65–70 min, was observed within a narrow *m*/*z* range (350–450), corresponding to highly hydrophobic compounds that only elute under strong organic conditions.

### 3.4. Overview of the Feature-Based Molecular Network for Ugandan Propolis

**Figure 4:**
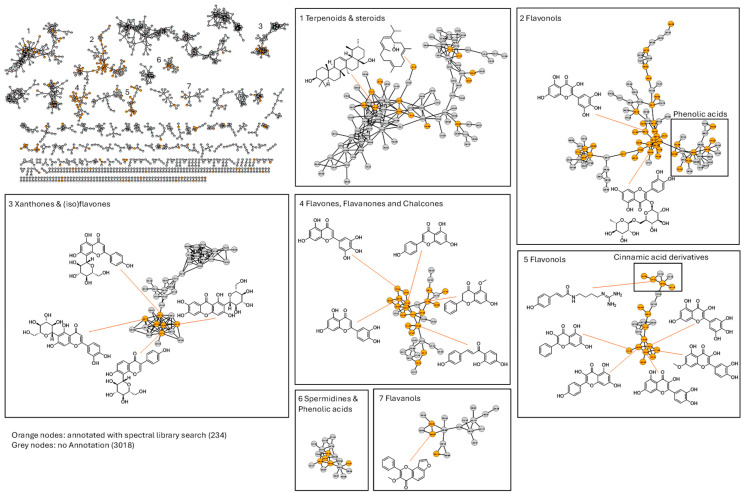
Molecular networking shows the distribution of detected compounds within all propolis samples. The network was generated from LC-MS/MS data with GNPS. The overview (upper-left corner) shows all molecular families with at least 2 connected nodes, using a cosine score threshold of 0.7. Seven clusters are highlighted, labelled with the precursor mass, and possible structures are shown that were matched with a spectral library. Orange nodes showed a spectral library hit within the molecular family. Grey nodes are not further classified.

A molecular network constructed in GNPS (link: https://gnps.ucsd.edu/ProteoSAFe/status.jsp?task=302cb78a6f4e49448bf6ac429d2bb8c1, accessed on 30 September 2025) illustrates how MS/MS-detected compounds are distributed across all propolis samples (Figure 4). In this network, each node corresponds to a unique molecular feature (consensus MS/MS spectrum), and edges link nodes with high spectral similarity (shared fragment ions), thereby grouping chemically related metabolites. The network, comprising 3252 nodes connected by spectral similarity, illustrates the chemical complexity of the samples. Out of these, 234 nodes (shown in orange in Figure 4) were tentatively annotated through spectral library matches, while 3018 nodes (in grey) remained unassigned due to the lack of library hits. The fact that over 90% of nodes remain unannotated might stem from the presence of numerous novel compounds or from incomplete or ineffective spectral libraries. We retained all molecular families with at least two connected nodes, yielding a highly interconnected map in which several distinct metabolite clusters could be identified.

### 3.5. Comprehensive Chemical Space Across Phytochemical Classes from Both Annotations

**Figure 5:**
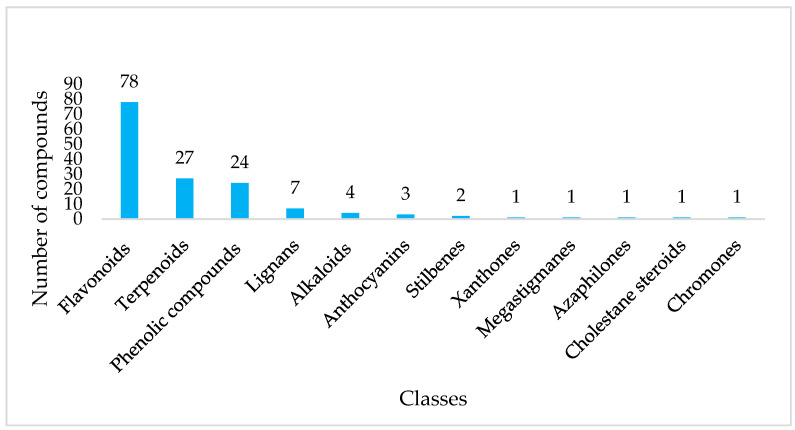
Distribution of compounds annotated by SIRIUS and GNPS across main phytochemical classes shows flavonoids as the most prevalent group with 78 compounds. Terpenoids and phenolic compounds follow with 27 and 24 compounds, respectively, while smaller amounts come from lignans, alkaloids, anthocyanins, stilbenes, xanthones, and other minor classes.

Analysis combining SIRIUS (see Supplementary File csv1) and GNPS (Supplementary File csv2) annotations showed that the Ugandan propolis LC-MS metabolome is primarily composed of flavonoids, accounting for most of the detected compounds (78 features). The chemical profile was then characterised by terpenoids (27) and phenolic compounds (24), along with smaller yet significant quantities of lignans, alkaloids, anthocyanins, stilbenes, xanthones, and different rare subclasses (Figure 5). Among the flavonoids, SIRIUS primarily identified glycosylated and highly oxygenated derivatives, including flavonoid-3-*O*-glycosides (6 features, RT 6.28–9.42 min, Mass 434.08–610.15 Da, all [M + H]^+^), flavonoid *C*-glycosides (4 features, RT 3.89–9.05 min, 434.12–772.20 Da, [M + H]^+^), methylated flavonoids (4 features, RT 13.54–29.29 min, 286.08–330.07 Da), 3′-hydroxyflavonoids (2 features, RT 14.23–14.65 min, 286.05–332.05 Da [M + H]^+^), flavonoid-7-*O*-glucuronides (2 features, RT 7.12–9.20 min, 508.11–538.12 Da, [M + H]^+^) and a flavonoid-3-*O*-glucuronide (quercetin 3-*O*-glucuronide; RT 8.16 min; 478.08 Da). This annotation method identified two distinct quercetin glycosides: quercetin-3-O-rutinoside (rutin) at RT 6.78 min (C_27_H_30_O_16_; *m*/*z* 610.15 [M + H]^+^) and quercetin-3-*O*-xyloside (reinutrin) at RT 9.42 min (C_20_H_18_O_11_; *m*/*z* 434.09 [M + H]^+^). Furthermore, the dataset identified the C-glycosylated flavonoid naringenin-6-C-glucoside (RT 7.25 min; C_21_H_22_O_10_; *m*/*z* 434.12 [M + H]^+^). In contrast, some of the flavonoid *C*-glycosides identified herein have not been previously reported in propolis, including the isoflavonoid C-glycoside puerarin (RT 13.27 min; C_21_H_20_O_9_; *m*/*z* 416.11 [M + H]^+^). For the methylated flavonoids group, four compounds exhibiting distinct *O*-methylation patterns (*O*-, 3-*O*-, and 7-*O*- substitutions) were annotated, namely dihydrotricin, 5,7,8-trimethoxyflavanone, 3,3′-*O*-dimethylquercetin, and sakuranetin. Only two compounds from the 3′-hydroxyflavonoid subclasses were tentatively identified, namely, luteolin (C_15_H_10_O_6_) and 3-*O*-methylmyricetin (C_16_H_12_O_8_). Other minor subclasses of flavonoids included prenylated flavanones, such as 6-prenylated flavanone (RT 29.61 min; C_20_H_20_O_6_; *m*/*z* 356.13) and 3′-prenylated flavanones (RT 42.60 min; C_26_H_30_O_5_; *m*/*z* 422.21).

The GNPS annotation library primarily matched aglycone flavonoids and related phenylpropanoid compounds, providing broad coverage of key classes relevant to propolis chemistry. Flavonols, with 21 features, were the most prevalent, with retention times (RT) ranging from 4.33 to 31.96 min and *m*/*z* values from 271.06 to 609.15 Da, mainly as [M + H]^+^ ions and, to a lesser extent, M + H. Several compounds showed intense spectral matches, each sharing at least seven fragment peaks with GNPS library entries. These included quercetin (RT 4.58 min; *m*/*z* 303.05; 11 peaks; [M + H]^+^), karanjin (RT 4.33 min; *m*/*z* 295.06; 10 peaks; [M + H]^+^), nictoflorin (RT 6.69 min; *m*/*z* 365.19; 10 peaks; [M + H]^+^), and rhamnetin (RT 11.59 min; *m*/*z* 317.07; 10 peaks; [M + H]^+^). Rutin (RT 4.61 min; *m*/*z* 611.16; 8 peaks; [M + H]^+^) also matched strongly and was notably annotated with the SIRIUS workflow. Flavones (19 features; RT 4.61–35.13 min; *m*/*z* 255.07–579.17) also span a broad polarity range, reflecting different hydroxylation and methylation patterns. This subclass included several compounds with strong spectral matches, such as apigenin (RT 9.12 min; *m*/*z* 271.06; M + H), tricetin (RT 10.04 min; *m*/*z* 303.05; [M + H]^+^), vitexin (RT 4.79 min; *m*/*z* 433.11; M + H), and orientin (RT 4.63 min; *m*/*z* 449.11; M + H), each sharing more than 10 fragmentation peaks with GNPS library spectra. Additionally, luteolin (RT 7.96 min; *m*/*z* 287.06; M + H), isovitexin (RT 4.80 min; *m*/*z* 415.10; M + H − H_2_O), and isoorientin (RT 4.61 min; *m*/*z* 393.06; M + H) shared nine peaks, supporting confident structural annotation. Flavanones (11 features; RT 5.50–32.28 min; *m*/*z* 195.09–435.13) include simple aglycones and more substituted derivatives typical of classical propolis.

Furthermore, GNPS annotations include isoflavones (4 features; RT 4.42–18.39 min), with puerarin, which was also annotated in SIRIUS, simple coumarins (3 features; RT 4.56–6.24 min), xanthones (2 features; RT 4.52–17.83 min), mangiferin (RT 4.52 min; *m*/*z* 423.09, 8 peaks, [M+H]^+^) was the only one annotated at different retention times and linear diarylheptanoids (2 features; RT 8.97–11.40 min), highlighting the broad structural and biosynthetic diversity of phenylpropanoid metabolites. This range underscores the GNPS library’s value as a comprehensive reference for analysing complex flavonoid and polyphenolic profiles in propolis.

Phenolic compounds were the third-largest chemical group, with 24 features classified in the SIRIUS “Most Specific Class” category. The main subgroup included hydroxycinnamic acids and their derivatives (7 features; RT 11.86–19.54 min; 440.20–673.30 Da; [M + H]^+^), followed by coumaric acids and their derivatives (4 features; RT 17.18–21.39 min; 583.27–802.36 Da). To the best of our knowledge, these coumaric acid derivatives are reported for the first time in propolis, indicating an expanded phenolic diversity in this matrix. Additionally, phenolic glycosides (3 features; RT 4.17–9.21 min; 354.10–492.15 Da) were identified, with biflorin and isobiflorin as the main representatives. The other subclass consisted of quinic acids and their derivatives, characterised by two main features. The most notable example is chlorogenic acid (RT 4.63 min; C_16_H_18_O_9_; *m*/*z* 354.095; [M + Na]^+^).

On the other hand, GNPS-based annotation expanded the diversity of phenolic compounds, particularly through the identification of hydroxycinnamic acid amide derivatives (6 features; RT 4.31–18.31 min; *m*/*z* 277.58–644.30; 6–8 shared peaks; [M + H]^+^). None of these hydroxycinnamic acid amides has been reported previously in propolis, indicating a potentially novel chemical subset within these samples. Terpenoids, totalling 27 compounds, were mainly annotated using the GNPS workflow, which yielded 24 class-level identifications. Diterpenoids, especially those of the labdane subclass, dominated this group, followed by primarane and isopimarane types. Other detected diterpenoid subclasses included podocarpane, abeoabietane, abietane, cembrane, kaurane, and phyllocladane skeletons, indicating a wide diversity of diterpenoids in the samples, many of which were identified for the first time in propolis based on our findings and the existing literature. Two well-known pimarane-type diterpenoid isomers, isopimaric acid (RT 34.23 min; *m*/*z* 303.23; 7 shared peaks; M + H) and sandaracopimaric acid (RT 9.43 min; *m*/*z* 303.05; 7 shared peaks; M + H), were confidently identified.

The SIRIUS annotation identified two significant diterpenoid features: 15-hydroxydehydroabietic acid (RT 35.54 min; C_20_H_28_O_3_; *m*/*z* 316.20; [M + Na]^+^) and a tigliane/ingenane-type acid (RT 41.85 min; C_32_H_42_O_8_; *m*/*z* 554.29; [M + H]^+^). Both are consistent with hydrophobic, resin-derived terpenoids that elute at late retention times. The tigliane-type diterpenoid represents a novel discovery within propolis, thereby expanding its known diterpenoid profile. Additional minor metabolite groups were identified, increasing the chemical diversity of the samples. Megastigmanes, along with several alkaloids such as reticuline and corynoxeine, and phenylethylamines such as synephrine, were annotated using GNPS. Stilbenes were detected using SIRIUS. A few rare subclasses, including azaphilones, chromones, and cholestane-type metabolites, appeared only once but still contributed significantly to the overall structural diversity of the metabolomic profile.

### 3.6. Chemometric Analysis of Ugandan Ethanolic Propolis Extracts from Different Locations

#### 3.6.1. PLS-DA Modelling of the Combined SIRIUS and GNPS-Annotated Metabolite Profiles

**Figure 6:**
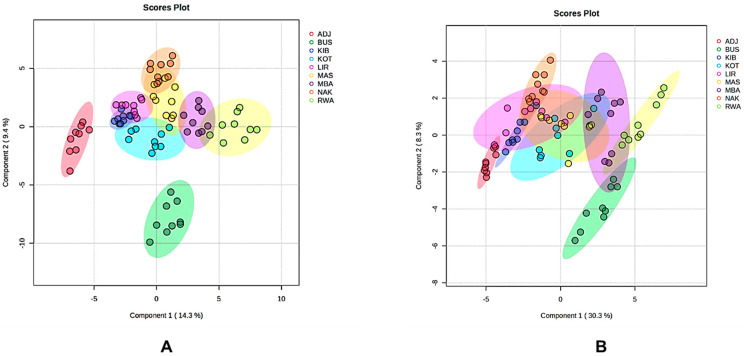
PLS-DA score plots: (**A**) Components 1 and 2 account for 14.3% and 9.4% of total variance for GNPS annotation; (**B**) Components 1 and 2 represent 30.3% and 8.3% of total variance for SIRIUS annotation.

Next, we applied PLS-DA in this study as a supervised technique to determine whether samples could be differentiated by location based on their chemical composition, thereby maximising group separation. Both GNPS-annotated metabolites and SIRIUS-annotated metabolic classes in the PLS-DA models reliably revealed clear chemical differences among the nine Ugandan propolis districts (Figure 6). These supervised multivariate models have been utilised to analyse the similarities and differences among metabolites by examining how they group into various clusters [19]. In the GNPS model, components 1 and 2 explained 14.3% and 9.4% of the variance, respectively (Figure 6A), leading to distinct clustering by district. ADJ samples formed tight clusters along component 2, whereas BUS, RWA, and KOT samples separated along component 1 of the score plot. An overlap was also observed near the centre among LIR and KIB along component 2, MBA and RWA along component 1, and between MAS and NAK samples along component 1, indicating consistent chemical profiles. The SIRIUS-based PLS-DA model effectively differentiated districts, with Component 1 accounting for 30.3% and Component 2 for 8.3% of the variance (see Figure 6B). ADJ consistently clustered with high intragroup similarity, sharing overlapping features with LIR samples along component 2. There was significant overlap near the origin among KOT, KIB, MAS, NAK, and LIR samples, indicating no differences within groups. Districts from western Uganda, such as BUS, MBA (which separated along component 2), and RWA (which separated along component 1), also showed overlaps, with no clear intragroup similarity among individual samples.

#### 3.6.2. Variable Importance in Projection (VIP) Features from GNPS and SIRIUS Annotations

**Figure 7:**
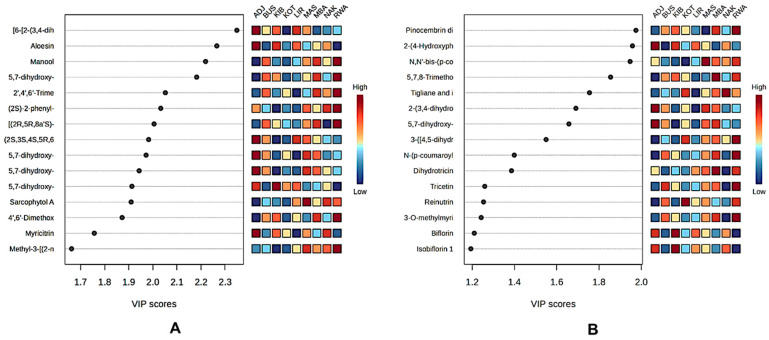
PLS-DA component 1 VIP scores (VIP > 1.0) for both annotations: (**A**) GNPS and (**B**) SIRIUS.

The VIP analyses (Figure 7) from both the SIRIUS- and GNPS-based PLS-DA models (See Figure 7) consistently show that Ugandan propolis exhibits strong district-specific metabolite signatures, with each region defined by a unique set of high-impact discriminant compounds [19]. The top discriminant in the SIRIUS-based model (Figure 7B) was pinocembrin dimethylether (VIP 1.97), which was highly abundant in RWA, making it a key marker for this region. ADJ was characterised by notably high levels of two complex flavonoid derivatives (VIP 1.96) and two related prenylated flavanones (VIP 1.69 and 1.66), highlighting its strong chemical uniqueness. MAS showed its highest enrichment with a phenolic acid, *N*,*N*′-bis-(p-coumaroyl)-N″-feruloyl spermidine (VIP 1.95), while MBA had elevated levels of 5,7,8-trimethoxyflavanone (VIP 1.90). A significant diterpenoid, tigliane and ingenane (VIP 1.80), was most abundant in NAK, accounting for its distinct position in the PLS-DA space. Several mid-ranking compounds also underscored regional differences in RWA (VIP > 1.20), including tricetin (VIP 1.30) and 3-*O*-methylmyricetin (VIP 1.24), making it one of the most chemically distinctive districts. KOT was characterised by reinutrin (VIP 1.25), and KIB by biflorin (VIP 1.21) and isobiflorin 1 (VIP 1.20).

The GNPS-based model (Figure 7A) similarly pinpointed a small but influential set of compounds that significantly distinguish geographic regions. In ADJ, the top two discriminants were [6-[2-(3,4-dihydroxyphenyl)-5,7-dihydroxy-4-oxochromen-3-yl]oxy]-glycosylated phenylpropanoate (VIP 2.34) and aloesin (VIP 2.26). Conversely, RWA exhibited strong enrichment of several high-ranking VIP features, such as manool (VIP 2.21), glycosylated chromen-4-one derivatives (VIP 2.18 and VIP 2.08), 2′,4′,6′-trimethoxychalcone (VIP 2.05), a pentamethyl spiro-furanonaphthalene derivative (VIP 2.00), and 4′,6′-dimethoxy-2′-hydroxychalcone (VIP 1.87).

#### 3.6.3. Heatmap Clustering for Chemical Marker Identification for GNPS-Annotated Phytochemical Groups

**Figure 8:**
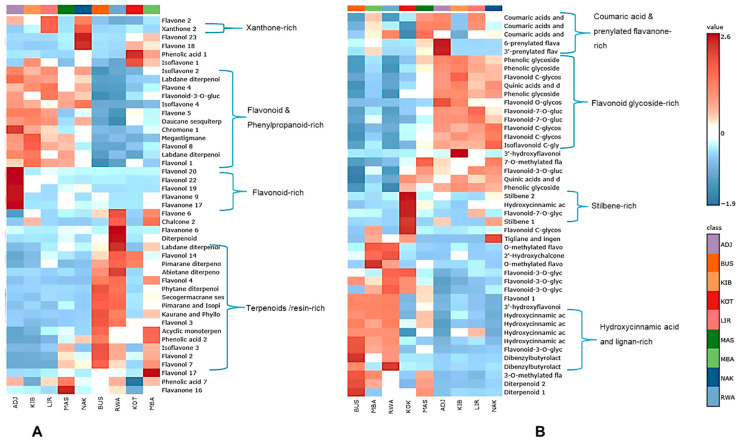
Shows heatmaps of the top 45 metabolite classes from both annotations, with rows representing compounds; columns represent samples grouped by district. Colour intensity indicates relative abundance (normalised scale). (**A**) GNPS and (**B**) SIRIUS.

The combined heatmap of the top 45 metabolites annotated by GNPS and SIRIUS revealed distinct, complementary chemical groups across Ugandan propolis (Figure 8). GNPS patterns (Figure 8A) showed that ADJ, KIB, and LIR formed a flavonoid- and phenylpropanoid-rich cluster, containing high levels of compounds like isoflavone 2, flavone 4 and 5, daucane sesquiterpenoids, megastigmane, flavonol 8 and 1, with ADJ additionally enriched in flavonol 20, 22, and 19, as well as flavanone 9 and 17. Conversely, BUS, RWA, and MBA exhibited a diterpenoid-dominant profile, with high abundance of flavonol 4, secogermacrane sesquiterpenoids, pimarane/isopimarane and kaurane diterpenoids, acyclic monoterpenoids, isoflavone 3, and labdane diterpenoids, forming a resin-rich chemical cluster. The kaurane-type diterpenoid 18,19-dihydroxykaur-16-en-6-one was annotated exclusively through GNPS, and a structurally related diterpenoid bearing the same kaurane (kaur-16-ene) backbone has previously been isolated from Saudi Arabian propolis [38]. These diterpenoids were previously isolated from *Croton tonkinensis* Gagnep. (Euphorbiaceae), indicating a probable botanical origin within this genus [39]. This is especially noteworthy because a related species, *Croton macrobothrys* Baill., has been recorded as a resin source for bees in Bushenyi (BUS) (see Supplementary Table S2). This suggests a potential connection between the diterpenoids and local *Croton* species. Other shared GNPS features linked specific districts: KOT and MBA (phenolic acid 1, isoflavone 1, flavone 18); NAK and LIR (xanthone 2, flavone 2); ADJ, LIR, MAS, RWA, MBA (phenolic acid 7); KIB, RWA, MBA (chalcone 2); MAS, RWA, MBA, BUS (flavonol 7, flavonol 2, isoflavone 3); and RWA alone, with high flavanone 6, broad diterpenoids, and labdane diterpenoids.

SIRIUS-annotated classes (Figure 8B) supported these results by revealing clustering at the metabolic class level. ADJ, KIB, LIR, and NAK showed elevated levels of phenolic glycosides, flavonoid *C*-glycosides, quinic acid derivatives, flavonoid-7-*O*-glycosides, and isoflavonoid C-glycosides, reflecting profiles rich in glycosylated phenylpropanoids. In contrast, BUS, MBA, and RWA had higher amounts of flavonoid-3-*O*-glycosides, flavonol 1,3-hydroxyflavonoids, and hydroxycinnamic acid derivatives, forming another SIRIUS-defined cluster enriched in aglycone and hydroxycinnamate-linked flavonoids. Additional class overlaps included BUS and MAS (3-*O*-methylated flavonoids, diterpene 2), MAS, ADJ, MBA (6-prenylated and 3-prenylated flavonoids), NAK, KOT, MBA (tiglanes/ingenanes diterpenoids), NAK, ADJ, KOT (stilbene 1; stilbene 2 in KOT), LIR, ADJ, MAS, MBA (coumaric acid derivatives), and NAK, LIR, KIB, MAS, KOT (quinic acid derivatives).

#### 3.6.4. Multivariate Differentiation of Propolis Samples Using Compounds Annotated by SIRIUS and GNPS

oPLS-DA analyses based on GNPS- and SIRIUS-annotated metabolites (Supplementary Table S3) revealed distinct chemical differences among the Ugandan propolis samples. It helps to resolve overlaps among sample groups, extending the separation achieved with PLS-DA (see Section 3.6.1) and enhancing the detection of subtle geographical variations among samples [19]. In our models, most district pairs showed a clear separation between groups, with T scores usually between 20% and 40%. Meanwhile, within-group variability stayed low, with orthogonal scores from 5% to 20%. These structural features had high R^2^Y values (≥0.94) and strong predictive ability (Q^2^ ≥ 0.90). Permutation tests (*n* = 20, *p* < 0.05) confirmed the models’ statistical significance. In the GNPS dataset (Supplementary Table S3A), the ADJ–RWA model ranked among the strongest, with a high T score (37.0%), low orthogonal variation (8.0%), and excellent model statistics (R^2^Y = 0.998; Q^2^ = 0.967) as shown in (Supplementary Figure S2A). A typical moderate GNPS model, MAS–RWA, exhibited a T score of 26.0%, orthogonal variation of 10.1%, and a moderate Q^2^ of 0.913, suggesting a partial but dependable separation between districts. In contrast, MAS–NAK was among the weakest GNPS models, showing a low T score (15.3%), greater orthogonal variation (10.2%), and the lowest predictive ability (Q^2^ = 0.730), indicating considerable overlap in their chemical profiles.

The SIRIUS-annotated dataset (Supplementary Table S3B) exhibited similar trends. The ADJ–RWA model remained one of the most effective, characterised by a very high T score (61.1%), a low orthogonal score (6.6%), and excellent performance metrics (R^2^Y = 0.998; Q^2^ = 0.986). These findings are shown in the SIRIUS OPLS-DA score plot and are confirmed by the permutation plot (Supplementary Figure S2B). A standard moderate SIRIUS model was MAS–RWA, achieving a T score of 37.7%, an orthogonal variation of 15.3%, and a Q^2^ of 0.924, indicating intermediate separation. The least effective SIRIUS model was LIR–NAK, which had the lowest T score (16.8%), the highest orthogonal score (19.7%), and minimal predictive ability (Q^2^ = 0.595), indicating poor differentiation between the two districts. The overall consistency between GNPS- and SIRIUS-based oPLS-DA results highlights the robustness of the identified chemical patterns. Both datasets reliably identify the strongest and weakest differences among districts. Pairs from ecologically distinct zones, such as ADJ and RWA, exhibit the most significant chemical differences. In contrast, pairs such as LIR–NAK and MAS–NAK, situated in similar ecological regions, show only slight separation. Using score plots, model validity metrics, permutation tests, and VIP analyses together provides a detailed view of spatial variation in propolis chemistry across Uganda.

#### 3.6.5. DSPC Network Analysis of SIRIUS- and GNPS-Annotated Compounds

The debiased sparse partial correlation (DSPC) networks (Supplementary Figure S3) created from annotated metabolites showed distinct yet complementary organisational patterns within the Ugandan propolis metabolome. In these networks, red edges indicate positive partial correlations, while blue edges represent negative or inverse relationships; the thickness of each edge reflects the strength of the correlation, with thicker edges signifying stronger direct connections [35]. These edge properties and node metrics, such as degree and betweenness centrality, help identify chemical modules, connected clusters, and metabolite classes that may indicate biosynthetic relationships [40]. The SIRIUS-derived DSPC network (Supplementary Figure S3A) showed a clear, organised structure mainly dominated by phenylpropanoids. It showed both strong and moderate positive links across phenolic subclasses. The strongest connections were between flavonoid-3-*O*-glycoside 3 and phenolic glycoside 4, and between diterpenoid 2 and dibenzylbutyrolactone lignan 1, highlighting tightly co-regulated metabolite pairs. Additional thin red edges linked various phenylpropanoid subclasses, including coumaric-acid derivatives, flavonoid glucuronides, flavonoid C-glycosides, O-methylated flavonoids, and quinic acid derivatives, forming a broad yet interconnected phenolic network. Centrality analysis revealed these structural patterns. Hydroxycinnamic acids and derivative 1 exhibited the highest degree (8) and betweenness scores (190.63), identifying them as the primary bridging metabolites that connect the key flavonoid and phenolic glycoside clusters to other phenolic subclasses. Peripheral phenolic groups, including prenylated chalcones and stilbenes, are linked by thinner red edges, while thin blue edges represent weaker or inverse relationships.

The GNPS-derived DSPC network (Supplementary Figure S3B) highlighted a key terpenoid–flavonoid axis, with the most interconnected regions marked by several thick red edges. The strongest correlations were identified between flavone 19 and flavonol 1, flavone 5 and flavone 4, isoflavone 1 and flavonol 3, isoflavone 1 and flavone 14, flavone 14 and phenolic acid 7, flavonol 6 and flavone 3, flavone 14 and carboxylic acid, phenolic acid 7 and phenolic acid 2, and oleanane triterpenoid 1 and naphthoquinone. These connections demonstrate strong co-occurrences among flavonoids, isoflavonoids, phenolic acids, and specific terpenoids. Although diterpenoids formed visible clusters within the network, flavonoid subclasses played key bridging roles. Nodes like flavone 2 (orientin), flavonol 3 (rutin), flavone 1 (isoorientin), and xanthone 1 (mangiferin) had the highest degree and betweenness values (20 and 16, respectively), emphasising flavonoids as the main connectors between diterpenoids and other compound classes such as phenolic acids, naphthoquinones, and chromones. The prominence of these flavonoids may reflect their shared biosynthetic origins in the phenylpropanoid pathway, their frequent co-occurrence in plant exudates, or their stability within the resin matrix. Peripheral groups, including monoterpenoids, phenylethylamines, and isolated diterpenoids, are connected by thin red or blue edges, signifying weaker links.

### 3.7. Selection of Potential Candidate Metabolites for In Silico Antibacterial Activity

#### 3.7.1. Identification of Chemical Markers Without Previously Reported Antibacterial Activity Using a Heatmap

**Figure 9:**
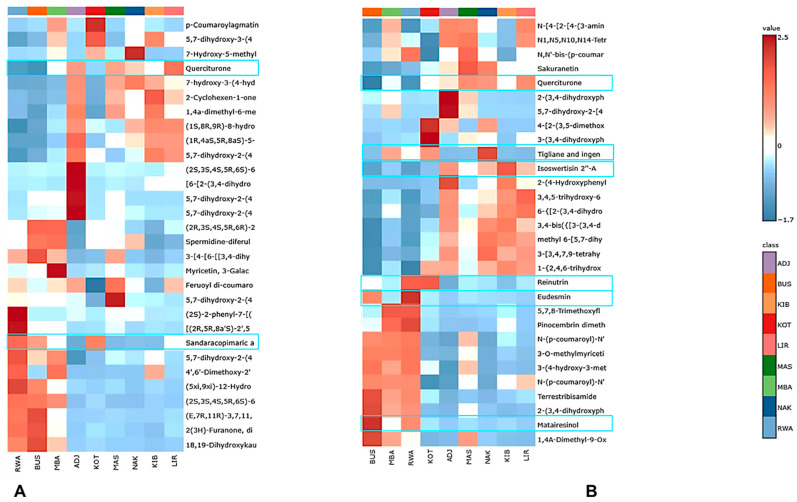
Shows heatmaps of the metabolite markers from both annotations with no reported antibacterial activity, with rows representing compounds; columns represent samples grouped by district. Colour intensity indicates relative abundance (normalised scale). (**A**) GNPS and (**B**) SIRIUS. The cyan boarder lines indicates the selected markers for in silico antibacterial activity.

To discover novel putative antibacterial candidate compounds from Uganda’s unexplored propolis, we systematically screened the combined GNPS and SIRIUS metabolite datasets. This step is important because propolis contains hundreds of compounds, many of which are chemically known but pharmacologically underrepresented, and prioritising such molecules is critical for drug discovery from complex natural mixtures. Using comprehensive literature searches in PubMed, Google Scholar and Scopus, we identified a total of twenty-eight (28) compounds with known antibacterial activity (Supplementary Table S4), covering multiple chemical classes (flavonoids, diterpenoids, lignans, alkaloids, chromones, and phenolic acids). These known antibacterial phytochemicals were excluded to focus the analysis on chemically abundant yet biologically unexplored metabolites, enabling the discovery of promising new antibacterial leads rather than rediscovering known ones. We then applied heatmap-based abundance profiling to identify probable biomarkers that lack known antibacterial activity, as shown in the GNPS- and SIRIUS-based heatmaps (Figure 9), revealed a diverse set of compounds with specific, location-dependent abundance patterns. Querciturone emerged as the most reliable biomarker, consistently detected in both heatmaps and with high levels in LIR, MAS, NAK, and ADJ. Its frequent appearance across multiple platforms and sites highlights its significance. Sandaracopimaric acid was consistently found in KOT, BUS, and RWA, creating a unique two-site pattern that chemically distinguishes these districts. Similarly, tigliane and ingenane diterpenoids were especially common in NAK, with some presence in KOT and MBA, making them strong chemotaxonomic markers and potential antibacterial agents. Multiple flavonoid derivatives provided equally insightful information. Isoswertisin 2″-acetate showed a clear clustering in LIR, KIB, NAK, and ADJ, while reinutrin was notably more prevalent in KOT and RWA. These compounds, from less-studied flavonoid subclasses, have unique structures suitable for in silico screening. The lignans, matairesinol and eudesmin, were highly enriched in RWA and BUS, respectively, forming distinct abundance clusters and expanding the list of marker compounds. These compounds were selected based on their distinctive heatmap clustering, high relative abundance, presence in at least two districts, and the lack of prior reports of antibacterial activity.

#### 3.7.2. Molecular Docking Analysis

Molecular docking predicts how a ligand fits in a receptor’s binding site to form a stable complex, estimating binding affinity with scoring functions and assessing interaction energies [30]. In our study, we observed simulations that revealed distinct structural differences in how the marker compounds, namely reinutrin, isoswertisin-2″-acetate, querciturone, sandaracopimaric acid, tigliane/ingenane diterpenoid, matairesinol, and eudesmin, interact with various Gram-positive and Gram-negative bacterial targets. Reinutrin showed very high predicted antibacterial activity by extensively interacting with critical enzymatic residues in both *Streptococcus mutans* (3AIC) and *Klebsiella pneumoniae* (6T77). In *S. mutans*, it achieved a binding energy of −10.9 kcal/mol (Table 1), docking deeply into the glucansucrase (an enzyme that converts sucrose into glucans, driving dental plaque formation) active site and forming multiple hydrogen bonds with residues His587, Asn862, Asn914, Asp593, and Asn481 (Supplementary Figure S4), key for sucrose recognition, acceptor alignment, and glucan-chain growth.

A similarly strong interaction was seen in *K. pneumoniae* (Supplementary Figure S5), with reinutrin showing a binding energy of −10.4 kcal/mol (Table 1). It formed multiple hydrogen bonds with Asp187, Lys155, Gly88, Asn86, Gly182, and Met188, supported by hydrophobic contacts with Thr186, Arg15, and Pro181. These residues define the enzyme’s substrate-recognition area, and their involvement suggests direct disruption of catalytic activity.

Querciturone, a flavonoid-3-*O*-glucuronide, also showed promising antibacterial activity predictions, with clear binding interactions (−9.6 kcal/mol) in the *E. coli* 1G27 active site (Supplementary Figure S6). The ligand binds in a compact, deeply embedded position, stabilised by a network of hydrogen bonds involving Ile44, Gln96, Cys90, Gly89, and Gly45, and additional reinforcement through hydrophobic interactions with Leu91, complemented by a salt bridge with His132 and a π–cation interaction with Arg97, which collectively enhance its overall binding affinity. Querciturone’s ability to interact with multiple anchoring residues across different protein targets explains its consistently high performance in docking assays, including a strong binding affinity with (3AIC) (−11.1 kcal/mol) as shown in Table 1.

#### 3.7.3. ADME Property Predictions and Drug-Likeness

ADME profiling via ADMETLab 3.0 [41] revealed diverse pharmacokinetic profiles for the studied phytochemicals, with positive traits and some limitations (Supplementary Table S5). All predicted Caco-2 permeability values were low (−6.593 to −4.754), indicating limited passive intestinal absorption. Although sandaracopimaric acid and eudesmin had higher scores, they did not meet the thresholds for effective GI uptake, as evidenced by their low MDCK permeability. P-gp interactions were minor; only the tigliane/ingenane diterpenoid and eudesmin were predicted to inhibit P-gp, suggesting that active transport does not significantly improve absorption. Plasma protein binding was high (69–97%), reducing the free drug fraction. Bioavailability predictions showed compound-specific variability. Reinutrin, Isoswertisin-2″-acetate, Sandaracopimaric acid, and Eudesmin were predicted to have oral bioavailability above 20%, whereas hydrophilic flavonoids, such as querciturone, had lower values. Metabolism predictions were favourable, with all compounds stable in human liver microsomes and exhibiting minimal inhibition of key cytochrome P450 enzymes, mainly CYP1A2 and CYP3A4. CYP2C19 inhibition was predicted only for matairesinol and eudesmin. Clearance and half-life data indicated moderate elimination, consistent with compounds of intermediate lipophilicity and limited polar metabolism. The calculated Quantitative Estimate of Drug-likeness (QED) scores ranged from approximately 0.22, indicating poor drug-likeness, to around 0.77, indicating good drug-likeness. Compounds like sandaracopimaric acid, matairesinol, and eudesmin showed QED values toward the higher end of this spectrum, suggesting they possess more favourable physicochemical properties for drug development. The ADME results show that although the compounds have good metabolic stability, low CYP inhibition, and acceptable drug-like properties, their limited intestinal permeability and high plasma protein binding pose pharmacokinetic challenges for systemic antibacterial use.

#### 3.7.4. Predicted Toxicological Profiles of the Candidate Compounds

The toxicity assessment using the ProTox-II platform provided insights into the predicted safety of phytochemicals, including evaluations of acute toxicity, organ toxicity, genotoxicity, immunotoxicity, endocrine effects, and stress pathways. Several compounds ranged from low to moderate predicted toxicity (Supplementary File csv3). Reinutrin and querciturone had LD_50_ values of 5000 mg/kg (Class 5), indicating low toxicity. Isoswertisin-2″-acetate had a lower LD_50_ of 832 mg/kg (Class 4), suggesting moderate toxicity. Sandaracopimaric acid, had the highest tolerability with an LD_50_ of 20,000 mg/kg (Class 6). The tigliane-ingenane diterpenoid with an LD_50_ of about 50 mg/kg (Class 2) showed high toxicity and was unsuitable for further development. Organ toxicity predictions highlight concerns for specific compounds. Reinutrin has a 0.74 probability, indicating borderline nephrotoxicity risk. In contrast, isoswertisin-2″-acetate triggered predictions of nephrotoxicity (0.85), with elevated carcinogenicity (0.71), mutagenicity (0.78), and immunotoxicity (0.81), raising safety concerns. Querciturone exhibits low toxicity probabilities, with carcinogenicity at 0.63, mutagenicity at 0.90 (inactive), and hepatotoxicity at 0.79 (inactive), indicating a better safety profile. Endocrine disruption assessments indicate that all compounds are inactive at the androgen receptor, oestrogen receptor, aromatase, and PPAR-γ, suggesting minimal endocrine risks. Stress-response pathway predictions were optimistic: compounds showed inactivity in the Nrf2/ARE, HSE, and p53 pathways, with probabilities mostly above 0.85. Mitochondrial membrane potential disruption was unlikely; querciturone’s probability was 0.63, but still non-activating. Toxicity predictions show most compounds are safe early on. Reinutrin, querciturone, and sandaracopimaric acid are top due to their low toxicity and lack of genotoxicity, endocrine effects, or stress-activation effects. Conversely, isoswertisin-2″-acetate, with moderate acute toxicity and high risks of carcinogenicity, mutagenicity, and immunotoxicity, should be deprioritised.

## 4. Discussion

From our HPLC-DAD chromatogram (Figure 1) and the overlay of the extracted ion chromatogram (EIC) from LC-MS analysis (Figure 2), we can clearly conclude that the elution patterns are unique among the propolis extracts. A comparable profile was observed earlier in the HPLC-DAD chemical profiling of propolis, with numerous compounds identified over a total elution time of 80 min, predominantly phenolic acids and flavonoids [42]. This chromatogram shows that the gradient effectively separates compounds across a broad polarity range, confirming its suitability for detailed profiling, even LC-MS/MS analysis. This confirms the chromatographic uniqueness and compositional differences among propolis from various locations in Uganda, consistent with a recent study reporting similar patterns in volatile compounds in Ugandan propolis [19]. The densest cluster of precursor ions in the MS/MS scatter plot appears within *m*/*z* 200–500, suggesting that low- to medium-mass phenolics like luteolin and flavonoid glycosides derived from quercetin and kaempferol could be predominant in the samples [43]. Few precursor ions were detected above *m*/*z* 800, suggesting that high-mass constituents are not prone to extensive fragmentation.

The molecular networking results clearly show the propolis’ chemical diversity, organising compounds into related families. Seven key clusters reveal a diverse range of natural products, mainly terpenoids and polyphenols. Spectral library annotation (orange nodes) identifies representative compounds, aiding classification. Notably, flavonoid clusters (2, 4, 5, 7) confirm flavonoids and phenylpropanoids as core components, consistent with propolis’ polyphenol profile. Clusters for terpenoids (1), xanthones/isoflavones (3), and spermidine derivatives (6) indicate diverse plant sources. These findings demonstrate molecular networking’s ability to profile complex natural products, group metabolites, and guide the annotation of known and new compounds, supporting targeted isolation and further research [44].

Beyond the well-established metabolites identified by SIRIUS, the detection of additional, structurally related flavonoid glycosides suggests the presence of previously unreported or rare glycosylated flavonoids in propolis, thereby enriching current understanding of its chemical diversity. The occurrence of rutin and reinutrin, previously found in ethanolic extracts of Portuguese [43], Moroccan [45], and Turkish propolis [46], highlights the widespread occurrence and chemotaxonomic significance of quercetin-based glycosides across different regions. The annotation of flavonoids with a C-glycosidic bond implies that, such as puerarin, could be due to the ability of honeybees to forage on plant species such as *Clematis* selectively [47]. The method further revealed significant structural differences among flavonoid glucuronides, particularly in the position of glucuronic acid attachment. For example, metabolites such as querciturone (C_21_H_18_O_13_; [M + H]^+^) have glucuronyl groups bonded at the 3-*O* position, along with evidence of 7-*O*-glucuronidation in flavonoids. Importantly, querciturone has been reported previously in Portuguese propolis [43], suggesting it occurs across various propolis chemotypes. Moreover, sakuranetin (RT 13.54 min; C_16_H_14_O_5_; *m*/*z* 286.08 [M + H]^+^), previously reported in Brazilian green propolis, is known to originate from botanical sources such as *Prunus* spp. and *Artemisia* spp. This association supports the role of plant resins from these taxa in shaping propolis’s chemical profile [48,49,50]. While 3-*O*-methylmyricetin has not been reported in propolis before, luteolin has been found in samples from regions such as Mexico and Turkey [51,52], indicating its widespread presence across various propolis types. The 2′-hydroxychalcone group was also present, with pinocembrin dimethyl ether (RT 31.99 min; C_17_H_16_O_4_; *m*/*z* 284.11) as an example, whose isomer (pinocembrin methyl ether) was reported in propolis from the United Kingdom [53]. Although these broader classes of natural products have been reported in propolis from various regions, the specific compounds identified in our study have not been previously documented. Prenylated flavonoids are present in propolis from areas such as Japan (Okinawa) [54], Taiwan [55], Brazil [56], the Solomon Islands [57], and Thailand [58]. Notably, Omani propolis contains prenylated flavonoids derived from plants such as *Azadirachta indica*, *Acacia nilotica*, and *Mangifera indica* [59]. This distribution, which differs by source, highlights the ecological significance and chemotaxonomic diversity of prenylated flavonoids across various types of propolis worldwide.

The flavonols detected by the GNPS annotation technique, which have been reported in both Brazilian and Portuguese propolis [43,56]. Additional well-matched flavonoids included myricetin (RT 7.96 min; *m*/*z* 319.04; 8 peaks; [M + H]^+^), reported at high concentrations in Polish propolis [60], and astragalin (RT 9.62 min; *m*/*z* 449.12; 8 peaks; [M + H]^+^), which, to our knowledge, is reported here for the first time in propolis. Kaempferol (RT 6.43 min; *m*/*z* 493.10; 7 peaks; [M + H]^+^), previously identified in Brazilian propolis [61], and galangin (RT 31.96 min; *m*/*z* 271.06; 7 peaks; [M + H]^+^), a recognised marker of poplar-type propolis from temperate regions such as Europe, South America, some parts of Africa (South Africa), China, among others, was also detected [62,63,64,65]. Tricetin and luteolin were reported in different bee products, such as European honey made from *Eucalyptus* species [66], which might also be the source of Ugandan propolis, specifically from known species like *Eucalyptus camaldulensis* Dehn [19] and bee pollen (for only tricetin) from Brazilian *Mimosa gemmulata* pollen grains, a known composition of propolis [67]. Recently, studies on ethanolic extracts of Thai and Turkish propolis identified vitexin and apigenin [68,69]. Notably, several other members of this subclass, with similarly high levels of shared fragment ions, are reported here for the first time in propolis, broadening the known chemical diversity of this flavonoid group. Most of the tentatively identified compounds in the flavanone subclass had more than seven shared fragment peaks, with the majority of them being new to the propolis chemical space; however, hesperidin (RT 9.61 min; *m*/*z* 611, 14 peaks, [M+H]^+^) has been majorly reported in Polish propolis [60] and alpinetin (RT 24.59 min; *m*/*z* 271.10, 7 peaks, M + H) in Chinese poplar-type propolis [70].

Furthermore, phenolic glycosides (3 features; RT 4.17–9.21 min; 354.10–492.15 Da) were identified, with biflorin and isobiflorin as the prominent representatives in the phenolic compound group, which was strongly annotated by SIRIUS. These compounds have been previously isolated from Cameroonian propolis [71], suggesting that certain phenolic glycosides may be common to propolis from different geographical regions. Additionally, chlorogenic acid is widely documented in various propolis chemotypes, with numerous reports in water and ethanolic extracts of Brazilian green propolis [56,72,73,74] and Turkish samples [75,76], emphasising its frequent occurrence in propolis rich in caffeoylquinic acid derivatives. In GNPS, the phenolic amide *p*-coumaroylagmatine, has previously been isolated from *Albizzia julibrissin* Durazz [77], suggesting a potential connection to the botanical resin bees visit. This observation is particularly relevant because beekeepers at multiple sampling sites reported similar species, *Albizia coriaria* Welw., *Albizia grandibracteata* Taub., *Albizia gummifera* (J.F. Gmel.) C.A. Sm., *Albizia lebbeck* (L.) Benth., and *Albizia zygia* (DC.) J.F. Macbr. as plant sources associated with propolis collection (see Supplementary Table S2). This agreement between chemical signatures and ethnobotanical observations strengthens the evidence that *Albizia*-derived resins could meaningfully contribute to the chemical profile of Ugandan propolis.

In addition to diterpenoids, other subclasses annotated in GNPS comprised secogermacrane sesquiterpenoids, acyclic monoterpenoids, and a pentacyclic triterpene, collectively characteristic of non-polar resin components often linked to coniferous plant sources [19]. Diterpenic acids are well-known components of propolis; for instance, isopimaric acid has been found in both Brazilian and Mediterranean (Greek) propolis [44,78,79,80] and sandaracopimaric acid has been identified in samples from Saudi Arabia [81]. Beyond the main diterpenoid subclasses, the analysis detected sarcophytol A, a distinctive cembrane diterpenoid. Its presence might indicate contributions from stored propolis, where fermented pollen or aged resin fractions can introduce cembranoid compounds into the overall chemical profile [82]. The dataset also identified manool, a labdane-type diterpenoid previously found in both Tunisian and Brazilian propolis [83,84], implying that labdane diterpenes may be a common chemical feature across different geographic propolis types. The only triterpenoid found was uvaol, a pentacyclic triterpene also present in Brazilian propolis [85], supporting its potential role as a marker of triterpenoid-rich plant resins that contribute to propolis formation. The tigliane-type diterpenoid, annotated only in SIRIUS, represents a new finding in the chemical space of propolis, thereby expanding its known diterpenoid profile. Compounds belonging to this class are well documented in *Euphorbia* species and *Croton* spp., both members of the Euphorbiaceae family [86,87]. Surprisingly, these species are similar to what beekeepers told us during our raw sample collection, which included *Euphorbia tirucalli* L. and *Croton macrobotrys* Baill. as resin-producing plants frequently visited by bees (see Supplementary Table S2), suggesting their potential contribution to propolis diterpenoid content.

Smaller subclasses were also identified, including dibenzylbutyrolactone lignans, with two significant compounds noted. Matairesinol was recognised both by SIRIUS (RT 23.31 min; C_20_H_22_O_6_; *m*/*z* 358.14; [M + H]^+^) and GNPS (RT 7.66 min; *m*/*z* 359.15; 11 shared peaks; [M + H]^+^) and has been previously found in hydroalcoholic extracts of Brazilian propolis [79]. The second lignan, eudesmin (RT 30.86 min; C_22_H_26_O_6_; *m*/*z* 386.17; [M + H]^+^), was also detected, as reported for Canary Islands (Spain) propolis [88]. Furthermore, GNPS identified a furanoid lignan named lariciresinol (RT 14.45 min; *m*/*z* 721.33; [2M + H]^+^), which has also been previously found in three bee products, i.e., honey, pollen, and propolis, from Brazilian stingless bees [89].

The combined analysis of the SIRIUS and GNPS datasets using retention times, accurate masses, adduct behaviour, and class-level fragmentation matches shows that Ugandan ethanolic propolis extracts have a highly diverse and complex metabolome. The chemical profile is mainly composed of flavonoids, including both heavily glycosylated forms and classical aglycones, along with significant amounts of phenolic acids and their derivatives, as well as a variety of resin-derived terpenoids. These key groups are supplemented by lignans, alkaloids, megastigmanes, and several rare, specialised metabolites, which, although less abundant, broaden the profile’s structural diversity. This extensive chemical diversity highlights the multiple botanical sources of Ugandan propolis. It indicates the variety of plant resins collected by bees across the sampled districts, showcasing the ecological and chemotaxonomic complexity of this natural product.

PLS-DA plots (see Figure 6) consistently differentiate districts, confirming a clear geographical pattern in Ugandan propolis’ chemical signatures. This segregation of chemical compounds within these samples and annotations, which contributes to their similarities in cases of overlap and differences within samples with tight, isolated clusters, may be attributed to bees foraging on common botanical species across various locations [19]. Several resinous plant species documented in our sample collection were noted across different regions. For instance, beekeepers from districts in western Uganda (BUS, MBA, and RWA) mentioned *Hesperocyparis lusitanica* (Mill.) Bartel (Cupressaceae) and *Melaleuca citrina* (Curtis) Dum. Cours (Myrtaceae). *Erythrina abyssinica* Lam. ex DC (Fabaceae) was observed in MAS and NAK, while *Mangifera indica* L. (Anacardiaceae) and *Eucalyptus camaldulensis* Dehn. (Myrtaceae) were reported in nearly all locations, as detailed in Supplementary Table S2. The VIP analyses (Figure 7) revealed that RWA is the most chemically distinct district in both datasets. Additional notable markers included a bis-glycosylated chromen-4-one (VIP 1.91), distinguishing KIB; sarcophytol A (VIP 1.90), linked to MAS; myricitrin (VIP 1.75), characteristic of ADJ, present in the Myrtaceae and Fabaceae families, has been shown to exhibit various pharmacological effects, such as antioxidant, anti-allergy, anti-inflammatory, neuroprotective, antibacterial, and wound healing activities [90,91,92]. The agreement between the two annotation methods strengthens the reliability of the PLS-DA models and verifies the presence of distinct regional chemotypes in Ugandan propolis.

The combined GNPS–SIRIUS heatmap analysis (see Figure 8) reveals that Ugandan propolis splits into two primary chemical groups, rich in flavonoids and phenolic glycosides (ADJ, KIB, LIR, and NAK), which closely resemble the characteristic chemical profile of temperate-region propolis [43]. Interestingly, these districts do not have similar climates, suggesting that their grouping in the same chemical space may be due to comparable local beekeeping methods, especially the prevalence of growing or keeping resin- and nectar-producing plants, which affect the bees’ foraging options [14]. The second group of districts (BUS, RWA, MBA, and KOT) primarily included diterpenoids and compounds derived from resin. This indicates a distinctive chemotype associated with tropical resin-producing plants, with MAS at the centre and all chemical groups shared among them. This type of propolis is usually found in tropical areas, especially across Africa [93]. It contains terpenes (such as monoterpenes, sesquiterpenes, diterpenes, and triterpenes), stilbenes, flavonoids, and diterpenic resin acids [19,93]. These results suggest that in tropical regions such as Uganda, propolis cannot be strictly categorised by geographic boundaries because of substantial overlap in resin-producing plants across different landscapes. The oPLSDA models further indicated that regional vegetation and ecological gradients play a significant role in shaping metabolite profiles [19]. The DSPC networks supported the prediction of plausible biosynthetic relationships among the annotated metabolites. In SIRIUS, the observed connectivity pattern positions hydroxycinnamic acids as early intermediates in the biosynthesis of downstream phenylpropanoid-derived metabolites, such as flavonoids, lignans, coumarins, stilbenes, and glycosylated derivatives [94]. The GNPS DSPC network exhibited coordinated behaviour among terpenoids, phenolics, and flavonoids, aligning with their biosynthetic pathways, namely the mevalonate, shikimate, and phenylpropanoid pathways, respectively [56].

Molecular docking results ranked the two flavonoids, renuitrin and querciturone, as the best-predicted antibacterial candidates. Moreover, reinutrin’s polar interactions were further supported by π–π stacking with Tyr916 and Tyr610, as well as a stabilising hydrophobic contact with Asp588, collectively securing the molecule within the catalytic channel. These non-polar/hydrophobic bonds were also observed in a recent in silico study on green prenylated phenylpropanoids from green propolis against *S. mutans* [30]. Since glucansucrase is responsible for extracellular polysaccharide synthesis and biofilm formation [95], the occupation of these catalytic residues suggests that reinutrin could interfere with biofilm-related virulence mechanisms in *S. mutans*. Similarly, in docking studies with the *K. pneumoniae* (6T77) protein, Thr186 was identified as one of the residues that form hydrogen bonds with a propolis flavonoid ligand, indicating its possible importance in ligand binding [96]. The presence of high binding energies, hydrogen bonds, π–π stacking, and hydrophobic interactions across pathogens suggests that reinutrin targets key residues. This supports effective enzymatic inhibition and broad-spectrum antibacterial activity. On the other hand, these hydrogen-bond interactions observed in querciturone (Supplementary Figure S6) are crucial for ligand binding to the 1G27 enzyme [97]. This suggests a potential mechanism to inhibit its catalytic activity and reduce bacterial viability, although direct experimental evidence of growth inhibition has not yet been confirmed. Furthermore, its structural characteristics are consistent with known antibacterial mechanisms of polyhydroxylated flavonoids, including inhibition of bacterial enzymes by binding to deep pockets, disruption of DNA-processing proteins through aromatic stacking, and possibly damage to membrane integrity due to its balanced hydrophobic and hydrophilic features [98]. These qualities collectively make querciturone a promising antibacterial candidate with diverse modes of action, warranting further biochemical and microbiological investigation. The flavonoid glycoside (isoswertisin-2ʺ-acetate) showed only moderate binding strength, ranging from −6.3 to −10.1 kcal/mol (Table 1) across the proteins. This may be due to the compound’s structure, in which acetylating the sugar moiety reduces the number of hydrogen-bond donors and increases steric bulk [99]. This limits the ligand to shallower binding modes and lowers the number of stabilising interactions compared to reinutrin and querciturone. The ADME predictions showed that none of the chemical markers were predicted to cross the blood–brain barrier, which is advantageous for systemic antibacterial agents, as CNS penetration is unnecessary and could raise toxicity risks [100]. Drug-likeness filters showed variability; many met Pfizer and Golden Triangle rules, but some, including querciturone and tigliane-ingenane diterpenoid, failed Lipinski criteria (e.g., molecular weight or H-bond counts slightly too high) [100,101]. Although these compounds passed the ADME test, some candidates posed issues such as insufficient oral bioavailability. To improve this, strategies such as structural modifications, nanocarriers or non-oral methods, such as topical treatments, could help realise their antibacterial potential and support clinical development. The compounds were predicted to be safe, with toxicity values consistent with the general observation that many natural products exhibit lower toxicity than their synthetic analogues [102]. Although immunotoxicity models in ProTox-II demonstrate only modest predictive accuracy [103], any immunosuppressive alerts would affect the inclusion of any lead compound (s) for consideration of an antibacterial agent.

## 5. Conclusions

This study presents the first detailed LC–HR–QTOF–MS-based metabolomic profile of Ugandan propolis, combining untargeted metabolomics, computational annotation, chemometric analysis, and in silico antibacterial testing. It moves beyond previous GC–MS studies of volatile compounds, greatly expanding the known chemical diversity. The metabolome comprises flavonoids, phenolic acids and their conjugates, lignans, megastigmanes, and many diterpenoids, many of which are newly reported in propolis. Multivariate analysis revealed geographic differences among samples from nine districts, showing two main patterns: flavonoid- and phenylpropanoid-rich profiles in the northern hemisphere, and diterpenoid-rich profiles in the southern hemisphere of Uganda. This indicates that ecological and botanical diversity influence the chemical makeup and highlights LC–MS-based metabolomics for chemotaxonomic and geographic differentiation of African propolis. We recommend further structural confirmation of the putatively annotated metabolites using NMR. Molecular docking identified lesser-known compounds, such as polyhydroxylated flavonoids (reinutrin and querciturone), as promising antibacterial candidates with strong predicted binding affinities and acceptable drug-like properties, highlighting the need for confirmation through in vitro assays. Overall, the framework supports targeted isolation, validation, pharmacology, standardisation, value addition, and sustainable use of Ugandan propolis as an antibacterial resource.

## Figures and Tables

**Table 1:** Predicted binding energies (kcal/mol) of propolis-derived ligands and positive controls against six bacterial target proteins.

Ligand Name	Ligand ID	Proteins					
		1JII	1G27	6T77	1BOW	3AIC	1U1Z
Reinutrin	L2	−11.8	−8.4	−10.4	−7.1	−10.9	−9.8
Isoswertisin 2″-acetate	L3	−9.2	−7.2	−8.5	−6.3	−10.1	−9.7
Querciturone	L4	−9.3	−9.6	−10.4	−7.8	−11.1	−9.7
Sandaracopimaric acid	L6	−7.3	−6.4	−7.9	−6.0	−8.8	−9.0
tigliane/ingenane diterpenoid	L7	−7.5	−7.7	−8.3	−5.3	−8.2	−8.7
Matairesinol	L8	−9.0	−7.9	−8.3	−6.1	−8.0	−8.8
Eudesmin	L9	−7.8	−6.6	−7.6	−4.8	−8.2	−9.0
Ciprofloxacin		−8.0	−8.0	−8.0	−5.8	−7.9	−8.7
Vancomycin		66.9	93.2	22.6	178.4	44.6	109.2

## Data Availability

All data utilised in this manuscript are included in the Supplementary Files. The original datasets are available only upon request from the corresponding author.

## Supplementary Materials

The following supporting information can be downloaded at: https://www.mdpi.com/article/10.3390/metabo16020109/s1, Supplementary Figure S1: Workflow for the selection of candidate antibacterial chemical markers from Ugandan propolis. Supplementary Figure S2: ADJ-RWA best pairwise comparison. Supplementary Figure S3: Debiased sparse partial correlation (DSPC) networks for SIRIUS and GNPS. Supplementary Figures S4–S6: Molecular Docking Interactions. Supplementary File csv1: SIRIUS annotated compounds. Supplementary File csv2: GNPS annotated compounds. Supplementary File csv3: Toxicity predictions. Supplementary Table S1: Shows Climatic factors of the agroecological zones. Supplementary Table S2: Shows the plant species mentioned by the 27 beekeepers in the different agro-ecological zones. Supplementary Table S3: oPLS-DA analysis of GNPS and SIRIUS-annotated compounds. Supplementary Table S4: Shows compounds annotated from both methods with reported antibacterial activity. Supplementary Table S5: In silico ADME characteristics and medicinal chemistry descriptors for ligands.
