# Supplementary material for: Integrative LC-HR-QTOF-MS and Computational Metabolomics Approaches for Compound Annotation, Chemometric Profiling and In Silico Antibacterial Evaluation of Ugandan Propolis
Source: Metabolites. 2026 Feb 3;16(2):109. doi: 10.3390/metabo16020109 (PMC12942557; doi:10.3390/metabo16020109)
Supplement: Supplementary file 1 [file metabolites-16-00109-s001.zip › Supplementary Figure S1-Workflow for the selection of candidate antibacterial chemical markers from Ugandan propolis.pdf]

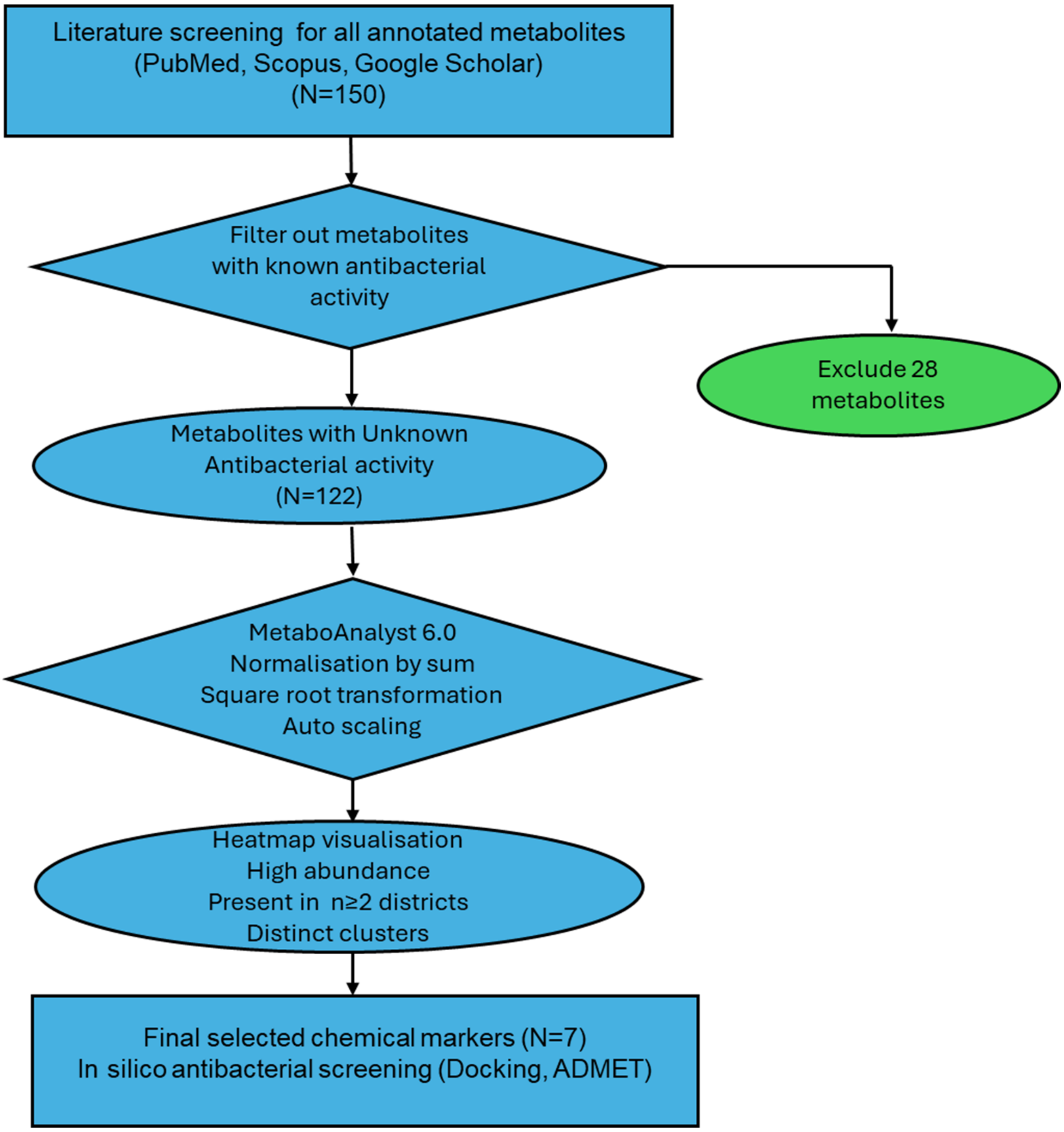

Supplementary Figure S1. Workflow for the selection of candidate antibacterial chemical markers from Ugandan propolis
