# Supplementary material for: Integrative LC-HR-QTOF-MS and Computational Metabolomics Approaches for Compound Annotation, Chemometric Profiling and In Silico Antibacterial Evaluation of Ugandan Propolis
Source: Metabolites. 2026 Feb 3;16(2):109. doi: 10.3390/metabo16020109 (PMC12942557; doi:10.3390/metabo16020109)
Supplement: Supplementary file 1 [file metabolites-16-00109-s001.zip › Supplementary Figure S3-Debiased sparse partial correlation (DSPC) networks for SIRIUS and GNPS.pdf]

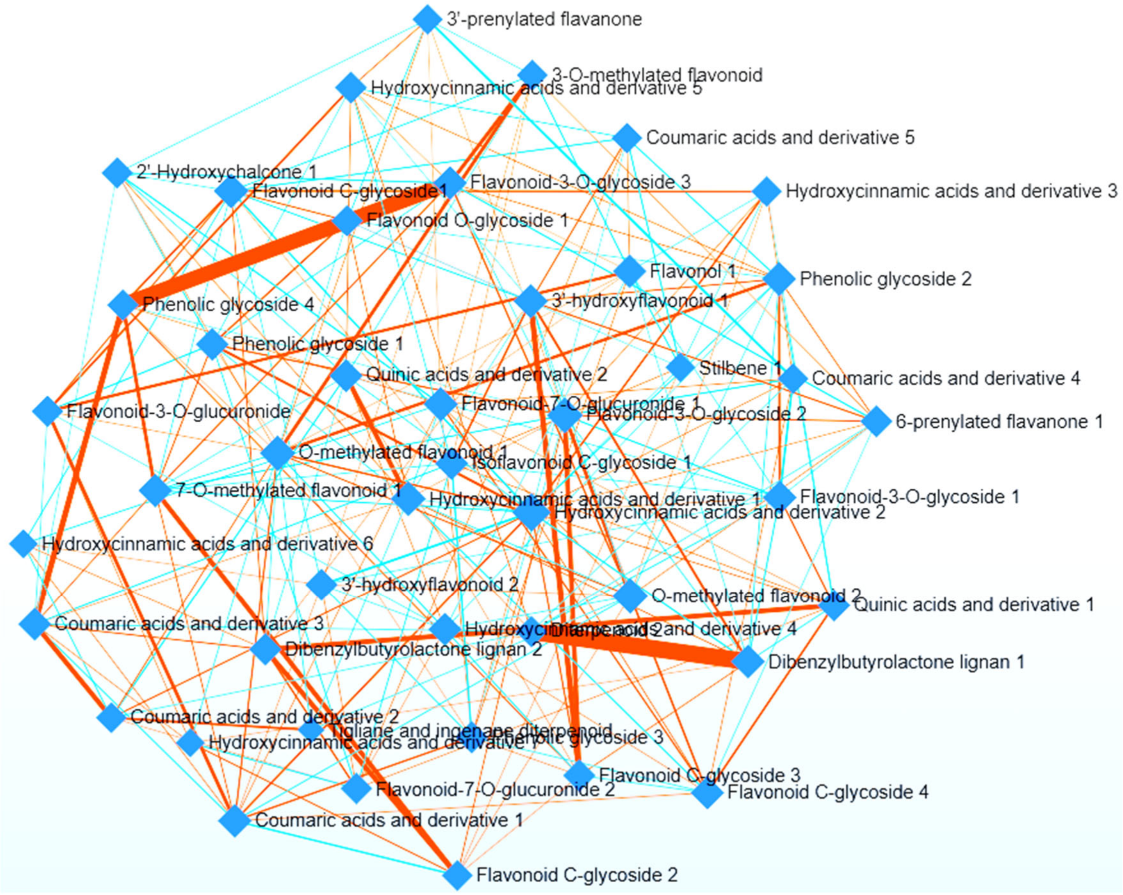

Supplementary Figure S3A. DSPC for SIRIUS annotated compounds classes

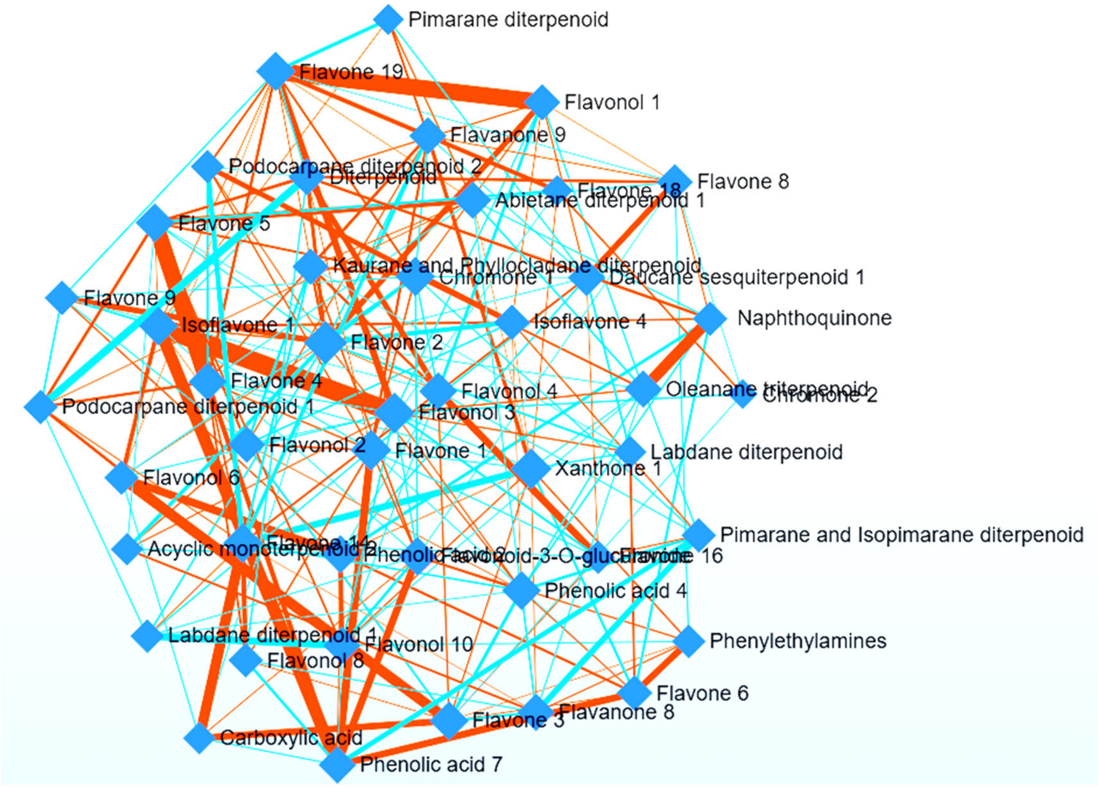

Supplementary Figure S3B. DSPC for GNPS annotated compounds classes
