# Supplementary material for: Integrative LC-HR-QTOF-MS and Computational Metabolomics Approaches for Compound Annotation, Chemometric Profiling and In Silico Antibacterial Evaluation of Ugandan Propolis
Source: Metabolites. 2026 Feb 3;16(2):109. doi: 10.3390/metabo16020109 (PMC12942557; doi:10.3390/metabo16020109)
Supplement: Supplementary file 1 [file metabolites-16-00109-s001.zip › Supplementary Figures S4-S6 Molecular Docking Interactions.pdf]

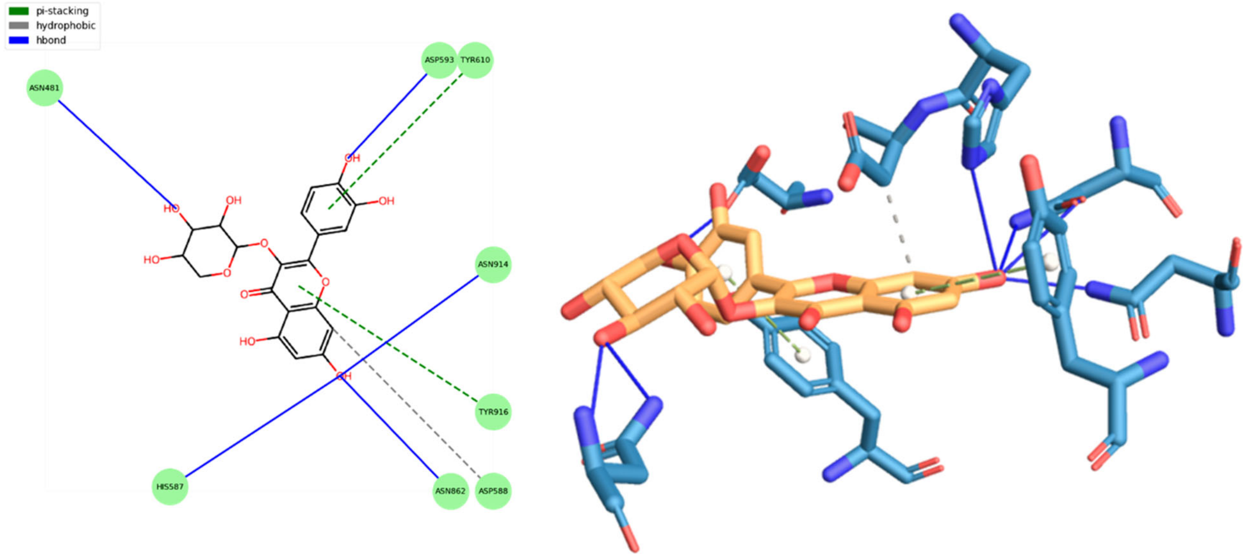

A B

Supplementary Figure S4. Molecular docking interactions of reinutrin with *S. mutans* (3AIC), (A): 2D representation, (B): 3D representation

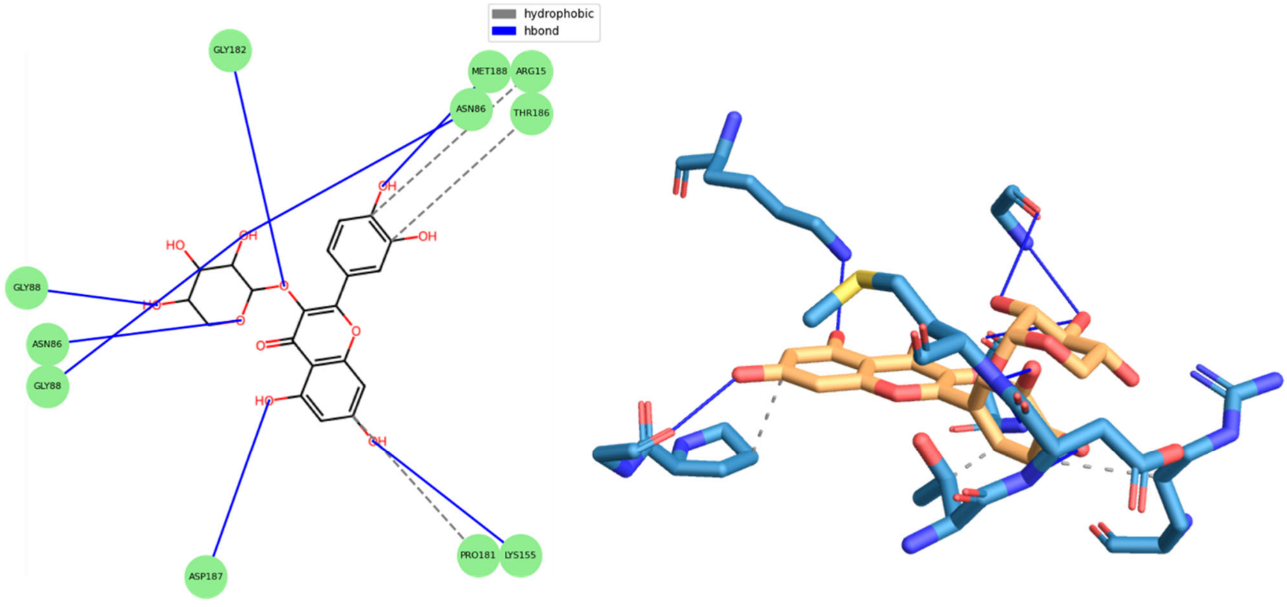

A B

Supplementary Figure S5. Molecular docking interactions of reinutrin with *K. pneumoniae* (6T77), (A): 2D representation, (B): 3D representation

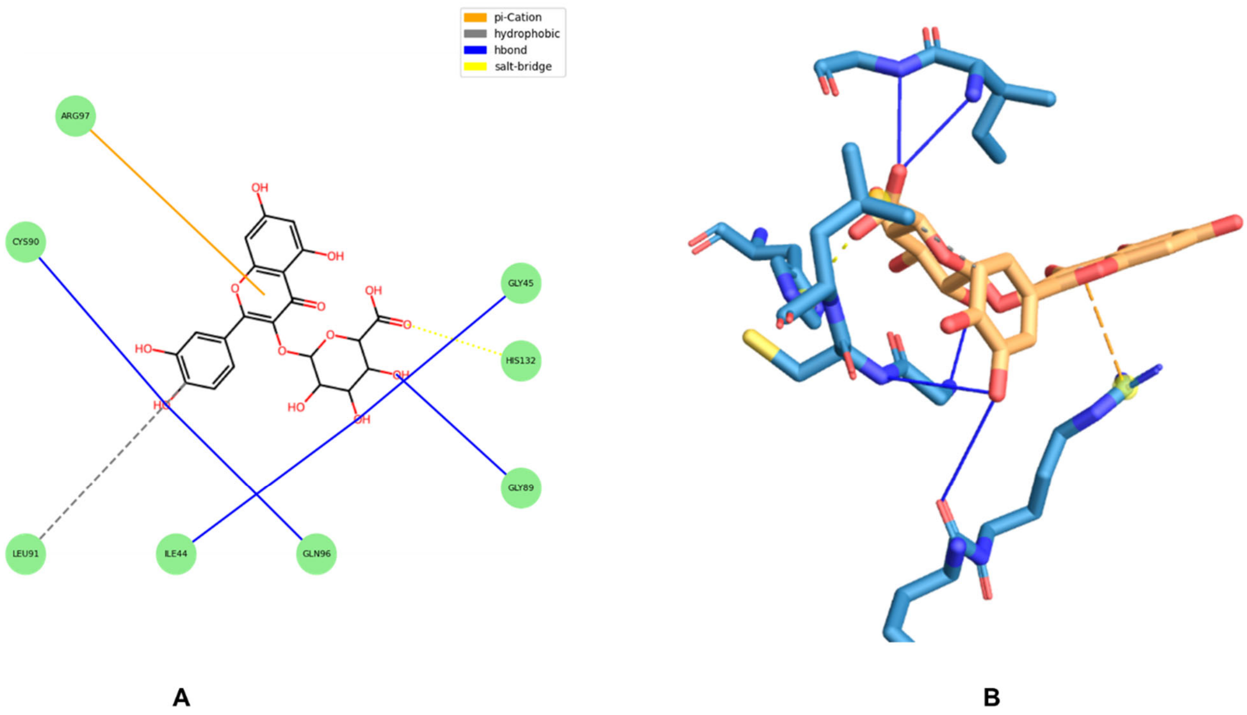

Supplementary Figure S6 Molecular docking interactions of quercituronе with *E. coli* (1G27), (A): 2D representation, (B): 3D representation
