# Supplementary material for: Integrative LC-HR-QTOF-MS and Computational Metabolomics Approaches for Compound Annotation, Chemometric Profiling and In Silico Antibacterial Evaluation of Ugandan Propolis
Source: Metabolites. 2026 Feb 3;16(2):109. doi: 10.3390/metabo16020109 (PMC12942557; doi:10.3390/metabo16020109)
Supplement: Supplementary file 1 [file metabolites-16-00109-s001.zip › Supplementary Table S1-Shows Climatic factors of the agroecological zones.pdf]

Supplementary **Table S1**. Shows Climatic factors of the agroecological zones

District	Agro-Ecological Zone	Approx. Area (km²)	Altitude (masl)	Dominant Vegetation	Major Soil Types	Geological Formation	Rainfall Pattern & Amount (mm/year)	Temperature Range (°C)	DOIs/links for references
Nakasongola	Lake Victoria Crescent	3,424	1,000–1,200	Wooded savanna with grasslands and forest patches	Ferralsols	Precambrian granites and gneisses	Bimodal: 1,000–1,200; Mar–May & Sep–Nov	17–30	https://doi.org/10.1080/21513732.2012.681070<br>https://doi.org/10.1002/ldr.2279
Masindi	Lake Albert Crescent	8,087	1,000–1,300	Tropical high forests, savanna woodlands, grasslands	Ferralsols, Acrisols	Precambrian granites and schists	Bimodal: 1,200–1,500; Mar–May & Sep–Nov	18–29	https://doi.org/10.1371/journal.pone.0263439<br>https://doi.org/10.1002/fsn3.490<br>https://hdl.handle.net/10568/54311<br>https://doi.org/10.1080/21513732.2012.681070<br>https://doi.org/10.1002/ldr.2279<br>https://oar.icrisat.org/10818/<br>https://doi.org/10.1038/s41561-024-01448-8

Bushenyi	Western Rangelands	3,949	1,200–2,200	Moist evergreen forests, savanna woodlands, grasslands	Nitisols, Ferralsols	Precambrian schists and quartzites	Bimodal: 1,200–1,800; Mar–May & Sep–Nov	15–27	https://doi.org/10.1371/journal.pone.0263439<br>https://www.ajol.info/index.php/ahs/article/view/6987
Kibuku	Eastern Rangelands	1,048	1,050–1,200	Savanna grassland with woodlands and wetlands	Ferralsols, Vertisols	Precambrian granites and gneisses	Bimodal: 1,100–1,300; Mar–May & Sep–Nov	18–30	https://doi.org/10.1371/journal.pone.0263439<br>https://doi.org/10.1038/s41598-022-11535-8<br>https://doi.org/10.1007/s40899-022-00604-5
Mbarara	Southern Rangelands	1,846	1,200–1,800	Savanna woodlands, grasslands, and wetlands	Ferralsols, Acrisols	Precambrian granites and schists	Bimodal: 1,000–1,200; Mar–May & Sep–Nov	16–28	https://doi.org/10.1371/journal.pone.0263439<br>https://doi.org/10.1002/ldr.2279
Lira	Northern Moist Farmlands	7,200	1,000–1,200	Moist savanna woodlands, grassland	Ferralsols, Luvisols	Precambrian granites and gneisses	Unimodal: 1,200–1,500; Apr–Oct	18–30	

				s, wetlands					
Adjumani	West Nile Farmlands	3,128	600–1,200	Savanna woodlands, grasslands, and wetlands	Ferralsols, Acrisols	Precambrian granites and gneisses	Unimodal: 1,000–1,200; Apr–Oct	20–32	https://doi.org/10.1371/journal.pone.0263439<br>https://doi.org/10.3389/fsufs.2025.1500012<br>https://doi.org/10.1144/gsjgs.130.3.0263
Rwampara	Southwestern Highlands	1,846	1,500–2,200	Moist evergreen forests, montane grasslands, and savanna woodlands	Nitisols, Ferralsols	Precambrian schists, quartzites, and gneisses	Bimodal: 1,200–1,800; Mar–May & Sep–Nov	14–25	https://doi.org/10.1371/journal.pone.0263439<br>http://academicjournals.org/journal/JENE/article-abstract/80936F152123
Kotido	Karamoja Drylands	3,612	900–1,200	Arid savanna grasslands with sparse woodlands and shrubs	Arenosols, Vertisols	Precambrian granites and schists, quartzite outcrops	Unimodal: 500–800; Apr–Sep	21–35	https://doi.org/10.1371/journal.pone.0263439<br>https://doi.org/10.3390/cli7030035<br>https://hdl.handle.net/10520/EJC119359
