# Supplementary material for: Integrative LC-HR-QTOF-MS and Computational Metabolomics Approaches for Compound Annotation, Chemometric Profiling and In Silico Antibacterial Evaluation of Ugandan Propolis
Source: Metabolites. 2026 Feb 3;16(2):109. doi: 10.3390/metabo16020109 (PMC12942557; doi:10.3390/metabo16020109)
Supplement: Supplementary file 1 [file metabolites-16-00109-s001.zip › Supplementary Table S2-Shows the plant species mentioned by the 27 beekeepers in the different agro-ecological zones.pdf]

Supplementary Table S2: Shows the plant species mentioned by the 27 beekeepers in the different agro-ecological zones

S/N	Species	Family	Plant parts	Districts	Freq
**1.**	*Albizia coriaria* Welw.	Fabaceae	Latex/Flowers	MAS & BUS	2
**2.**	*Albizia gribracteata* Taub.	Fabaceae	Latex/Flowers	MAS & LIR	4
**3.**	*Albizia gummifera* (J.F. Gmel.) C.A.Sm.	Fabaceae	Resins/Flowers	NAK	1
**4.**	*Albizia lebbeck* (L.) Benth.	Fabaceae	Resin	LIR	1
**5.**	*Albizia zygia* (DC.) J.F.Macbr.	Fabaceae	Resins/Flowers	NAK	3
**6.**	*Artocarpus heterophyllus* Lam.	Moraceae	Latex	MAS	2
**7.**	*Asystasia gangetica* (L.) T. & erson	Acanthaceae	Flowers	MAS	1
**8.**	*Azadirachta indica* A. Juss.	Meliaceae	Flowers	KIB	1
**9.**	*Balanites aegyptiaca* (L.) Delile	Zygophyllaceae	Resin	KOT	1
**10**	*Bidens pilosa* L.	Asteraceae	Flowers	MAS &KIB	3
**11**	*Cajanus cajan* (L.) Huth	Fabaceae	Flowers	MAS	1
**12**	*Calliandra calothyrsus* Meisn.	Fabaceae	Flowers	MAS, KIB, NAK, KOT LIR, MBA & RWA	10
**13**	*Carica papaya* L.	Caricaceae	Latex/Flowers	KIB & KOT	2
**14**	*Carissa edulis* (Forssk.) Vahl	Apocynaceae	Flowers	NAK	1
**15**	*Carissa spinarum L.*	Apocynaceae	Flowers	ADJ	1
**16**	*Citrus reticulata* Blanco	Rutaceae	Flowers	KIB	2
**17**	*Citrus sinensis* L.	Rutaceae	Flowers	KIB	3
**18**	*Coffea canephora* Pierre	Rubiaceae	Flowers	MAS, KIB, BUS, MBA, & RWA	9
**19**	*Combretum collinum* Fresen	Combretaceae	Resins/Flowers	MAS, NAK, & LIR	4
**20**	*Combretum molle* R.Br. ex G.Don.	Combretaceae	Resins/Flowers	NAK	3
**21**	*Commiphora africana* (A.Rich.) Engl.	Burseraceae	Resins	KOT	1
**22**	*Croton macrobothrys* Baill.	Euphorbiaceae	Resins/Flowers	BUS	1
**23**	*Elaeophorbia drupifera* (Thonn.) Stapf	Euphorbiaceae	Latex/Flowers	NAK	1
**24**	*Erythrina abyssinica* Lam. ex DC	Fabaceae	Resins/Flowers	MAS & NAK	2

25	*Eucalyptus camaldulensis* Dehn.	Myrtaceae	Resins/Flowers	MAS, KIB, NAK, BUS, MBA & RWA	13
26	*Euphorbia tirucalli* L.	Euphorbiaceae	Latex	KOT & RWA	2
27	*Ficus natalensis* Hochst.	Moraceae	Resins	BUS & RWA	3
28	*Ficus sycomorus* L.	Moraceae	Resins	KIB	1
29	*Grevillea robusta* A.Cunn. ex R.Br.	Proteaceae	Resins/Flowers	LIR	2
30	*Grewia mollis* Juss.	Tiliaceae	Flowers	NAK	1
31	*Hesperocyparis lusitanica* (Mill.) Bartel	Cupressaceae	Resins	BUS, MBA, & RWA	5
32	*Khaya anthotheca* (Welw.) C. DC.	Meliaceae	Latex	MAS	1
33	*Lantana camara* L.	Verbenaceae	Flowers	KIB, NAK, & LIR	5
34	*Mangifera indica* L.	Anacardiaceae	Resins/Flowers	MAS, KIB, MBA, NAK, BUS, & RWA	9
35	*Manihot esculenta* Crantz	Euphorbiaceae	Flowers	MAS	1
36	*Melaleuca citrina* (Curtis) Dum.Cours.	Myrtaceae	Resins/Flowers	BUS & MBA	3
37	*Melia azedarach* L.	Meliaceae	Resins	LIR	1
38	*Milicia excelsa* (Welw.) C.C.Berg	Moraceae	Resins	LIR	2
39	*Mitragyna rubrostipulata* (K.Schum.) Havil.	Rubiaceae	Resins/Flowers	BUS	1
40	*Moringa oleifera* Lam.	Moringaceae	Flowers	KIB	1
41	*Musa paradisiaca* L.	Musaceae	Latex /Flowers	LIR & RWA	3
42	*Ocimum tenuiflorum* L.	Lamiaceae	Flowers	MAS & NAK	2
43	*Persea americana* Mill	Lauraceae	Flowers	MAS, KIB, BUS & RWA	6
44	*Piliostigma thonningii* (Schumach.) Milne-Redh.	Fabaceae	Resins	LIR	2
45	*Pinus patula* Schiede ex Schltdl. & Cham.	Pinaceae	Resins/Flowers	MAS, BUS , LIR, MBA & RWA	7
46	*Podocarpus latifolius* (Thunb.) R.Br. ex Mirb.	Podocarpaceae	Latex	BUS	1
47	*Pseudomussaenda flava* Verdc.	Rubiaceae	Flowers	ADJ	1
48	*Psidium guajava* (L.)	Myrtaceae	Flowers	MAS, KIB, & RWA	2
49	*Ricinus communis* L.	Euphorbiaceae	Flowers	MAS	1

50	*Saccharum officinarum L.*	Poaceae	Others (Juice)	MAS & MBA	2
51	*Senna obtusifolia* (L.) HSIrwin & Barneby	Fabaceae	Flowers	ADJ	1
52	*Senna spectabilis* (DC.) H. S. Irwin & R. C. Barneby	Fabaceae	Flowers	MAS	1
53	*Sesamum indicum L.*	Pedaliaceae	Flowers	LIR	1
54	*Spathodea campanulata* P. Beauv.	Bignoniaceae	Flowers	NAK	1
55	*Tamarindus indica* L.	Fabaceae	Flowers	KIB	1
56	*Terminalia glaucescens* Planch.ex.Benth	Combretaceae	Resins	LIR	2
57	*Tithonia diversifolia* A. Gray.	Asteraceae	Flowers	MAS	1
58	*Vachellia hockii* (De Wild.) Seigler & Ebinger	Fabaceae	Resins/Flowers	NAK, LIR, MBA	4
59	*Vachellia seyal* (Delile) P.J.H.Hurter	Fabaceae	Resin	KOT	1
60	*Vachellia sieberiana* (DC.) Kyal. & Boatwr.	Fabaceae	Flowers	KOT	1
61	*Vitex doniana* Sweet	Lamiaceae	Resins/Flowers	NAK,LIR,	5
62	*Zizyphus abyssinica* Hochst	Rhamnaceae	Resins/ flowers	LIR	1
