# Supplementary material for: Integrative LC-HR-QTOF-MS and Computational Metabolomics Approaches for Compound Annotation, Chemometric Profiling and In Silico Antibacterial Evaluation of Ugandan Propolis
Source: Metabolites. 2026 Feb 3;16(2):109. doi: 10.3390/metabo16020109 (PMC12942557; doi:10.3390/metabo16020109)
Supplement: Supplementary file 1 [file metabolites-16-00109-s001.zip › Supplementary Table S3-oPLS-DA analysis of GNPS and SIRIUS-annotated compounds.pdf]

Supplementary Table S3A:oPLS-DA Analysis of GNPS-Annotated Compounds in Propolis Samples

T Score (%)	Orth. Score (%)	R^2Y	Q^2	Permutations (n = 20), $p < 0.05$	Pairwise Comparison
21.3	9.8	0.978	0.911	Q^2: 0.939, R^2Y:0.996	ADJ-KIB
29.6	13.0	0.977	0.941	Q^2: 0.968, R^2Y:0.996	ADJ-MBA
24.6	12.6	0.970	0.918	Q^2: 0.950,R^2Y:0.993	MAS-BUS
19.2	9.8	0.984	0.882	Q^2: 0.917,R^2Y:0.997	ADJ-NAK
16.8	10.6	0.933	0.816	Q^2: 0.885,R^2Y:0.991	LIR-NAK
37.0	8.0	0.985	0.967	Q^2: 0.978,R^2Y:0.998	ADJ-RWA
29.5	17.3	0.957	0.915	Q^2: 0.963,R^2Y:0.993	KIB-MBA
38.5	11.3	0.984	0.970	Q^2: 0.979,R^2Y:0.994	KIB-BUS
37.7	9.8	0.978	0.964	Q^2: 0.974,R^2Y:0.994	KIB-RWA
27.6	9.7	0.978	0.941	Q^2: 0.967,R^2Y:0.997	KOT-RWA
21.9	12.3	0.978	0.922	Q^2: 0.957,R^2Y:0.996	KOT-MAS
26.0	10.1	0.963	0.913	Q^2: 0.944,R^2Y:0.993	MAS-RWA
23.8	8.9	0.962	0.892	Q^2: 0.931,R^2Y:0.995	KOT-NAK
15.0	12.6	0.879	0.728	Q^2: 0.863,R^2Y:0.997	LIR-MAS
17.5	10.6	0.968	0.872	Q^2: 0.920,R^2Y:0.990	MAS-MBA
24.2	16.0	0.970	0.910	Q^2: 0.944,R^2Y:0.990	LIR-MBA
32.8	10.0	0.977	0.953	Q^2: 0.975,R^2Y:0.996	LIR-RWA
29.5	9.2	0.977	0.944	Q^2: 0.964,R^2Y:0.996	KOT-ADJ
26.7	10.6	0.983	0.945	Q^2: 0.967,R^2Y:0.996	KOT-BUS
29.6	12.8	0.969	0.942	Q^2: 0.976,R^2Y:0.995	KOT-KIB
24.6	12.4	0.968	0.926	Q^2: 0.960,R^2Y:0.995	KOT-LIR
21.7	14.7	0.978	0.921	Q^2: 0.957,R^2Y:0.995	KOT-MBA
16.8	11.7	0.958	0.836	Q^2: 0.901,R^2Y:0.995	LIR-ADJ
32.4	11.3	0.977	0.952	Q^2: 0.965,R^2Y:0.991	LIR-BUS
33.7	9.2	0.974	0.944	Q^2: 0.965,R^2Y:0.991	NAK-RWA
16.5	7.8	0.978	0.843	Q^2: 0.892,R^2Y:0.996	LIR-KIB
15.3	10.2	0.896	0.730	Q^2: 0.829,R^2Y:0.986	MAS-NAK
21.2	13.7	0.938	0.861	Q^2: 0.915,R^2Y:0.987	MAS-KIB
21.6	16.6	0.950	0.868	Q^2: 0.928,R^2Y:0.985	MBA-BUS
21.3	15.8	0.943	0.853	Q^2: 0.892,R^2Y:0.978	MBA-RWA
23.9	12.7	0.975	0.919	Q^2: 0.892,R^2Y:0.978	NAK-MBA
34.3	9.0	0.977	0.955	Q^2: 0.968,R^2Y:0.995	NAK-BUS
23.5	12.0	0.961	0.908	Q^2: 0.950,R^2Y:0.997	NAK-KIB
17.1	13.2	0.943	0.829	Q^2: 0.898,R^2Y:0.995	RWA-BUS
20.9	11.8	0.939	0.863	Q^2: 0.925,R^2Y:0.992	ADJ-MAS

Supplementary Table S3B: oPLS-DA Analysis of SIRIUS-Annotated Compounds in Propolis Samples

T Score (%)	Orth. Score (%)	R^2Y	Q^2	Permutations (n = 20), $p < 0.05$	Pairwise Comparison
32.3	14.7	0.981	0.961	Q^2: 0.972, R^2Y:0.990	ADJ-KIB
47.6	16.0	0.955	0.935	Q^2: 0.985, R^2Y:0.993	ADJ-MBA
32.9	13.8	0.959	0.924	Q^2: 0.953, R^2Y:0.991	MAS-BUS
23.6	18.5	0.881	0.827	Q^2: 0.949, R^2Y:0.992	ADJ-NAK
16.8	19.7	0.761	0.595	Q^2: 0.741, R^2Y:0.949	LIR-NAK
61.1	6.6	0.989	0.986	Q^2: 0.993, R^2Y:0.998	ADJ-RWA
45.8	17.0	0.941	0.917	Q^2: 0.974, R^2Y:0.989	KIB-MBA
45.9	11.7	0.994	0.985	Q^2: 0.992, R^2Y:0.998	KIB-BUS
54.3	8.4	0.989	0.984	Q^2: 0.991, R^2Y:0.997	KIB-RWA
38.6	15.7	0.963	0.938	Q^2: 0.973, R^2Y:0.990	KOT-RWA
26.6	11	0.948	0.916	Q^2: 0.968, R^2Y:0.989	KOT-MAS
37.7	15.3	0.954	0.924	Q^2: 0.953, R^2Y:0.986	MAS-RWA
25.5	12.5	0.967	0.920	Q^2: 0.964, R^2Y:0.992	KOT-NAK
20.3	21.3	0.677	0.578	Q^2: 0.813, R^2Y:0.989	LIR-MAS
21.2	21.5	0.923	0.823	Q^2: 0.924, R^2Y:0.984	MAS-MBA
38.4	18.8	0.931	0.904	Q^2: 0.966, R^2Y:0.986	LIR-MBA
49.3	11.9	0.971	0.963	Q^2: 0.984, R^2Y:0.994	LIR-RWA
37.3	11.3	0.979	0.966	Q^2: 0.986, R^2Y:0.996	KOT-ADJ
36.2	15.3	0.983	0.960	Q^2: 0.986, R^2Y:0.996	KOT-BUS
33.4	20.0	0.959	0.921	Q^2: 0.968, R^2Y:0.989	KOT-KIB
28.5	19.4	0.940	0.898	Q^2: 0.949, R^2Y:0.984	KOT-LIR
29.1	18.0	0.970	0.934	Q^2: 0.971, R^2Y:0.988	KOT-MBA
17.9	23.3	0.749	0.648	Q^2: 0.924, R^2Y:0.987	LIR-ADJ
44.6	12.6	0.980	0.970	Q^2: 0.981, R^2Y:0.993	LIR-BUS
52.1	5.1	0.989	0.981	Q^2: 0.985, R^2Y:0.997	NAK-RWA
18.1	21.6	0.919	0.805	Q^2: 0.889, R^2Y:0.987	LIR-KIB
23.4	17.3	0.785	0.710	Q^2: 0.868, R^2Y:0.989	MAS-NAK
31.2	18.6	0.812	0.760	Q^2: 0.905, R^2Y:0.984	MAS-KIB
29.5	17.6	0.866	0.826	Q^2: 0.955, R^2Y:0.986	MBA-BUS
26.4	22.5	0.853	0.792	Q^2: 0.914, R^2Y:0.979	MBA-RWA
39.8	14.6	0.958	0.930	Q^2: 0.973, R^2Y:0.992	NAK-MBA
46.5	7.7	0.985	0.975	Q^2: 0.985, R^2Y:0.995	NAK-BUS
27.8	16.1	0.952	0.917	Q^2: 0.952, R^2Y:0.988	NAK-KIB
17.6	21.0	0.922	0.809	Q^2: 0.900, R^2Y:0.988	RWA-BUS
31.8	17.4	0.865	0.821	Q^2: 0.948, R^2Y:0.989	ADJ-MAS
