# Supplementary material for: Integrative LC-HR-QTOF-MS and Computational Metabolomics Approaches for Compound Annotation, Chemometric Profiling and In Silico Antibacterial Evaluation of Ugandan Propolis
Source: Metabolites. 2026 Feb 3;16(2):109. doi: 10.3390/metabo16020109 (PMC12942557; doi:10.3390/metabo16020109)
Supplement: Supplementary file 1 [file metabolites-16-00109-s001.zip › Supplementary Table S4-Shows compounds annotated from both methods with reported antibacterial activity.pdf]

Supplementary Table S4: Shows compounds annotated from both methods with reported antibacterial activity

S/N	Compound	Class	Annotation	Key Publication DOI
1	Karanjin	Furanoflavonol	GNPS	10.1055/s-0042-105159
2	Mangiferin	Xanthone	GNPS	10.1007/s00203-025-04240-3
3	Quercetin	Flavonol	GNPS	10.1371/journal.pone.0134684
4	Rutin	Flavonol	GNPS & SIRIUS	10.3390/ijms252413684
5	Isoorientin	Flavone	GNPS	10.1093/jas/skad047
6	Orientin	Flavone	GNPS	10.3390/molecules30112300
7	Vitexin	Flavone	GNPS	10.1038/srep23347
8	Isovitexin	Flavone	GNPS	10.4014/jmb.2206.06007
9	Reticuline	Isoquinoline alkaloid	GNPS	10.1016/0378-8741(92)90058-y
10	Kaempferol	Flavonol	GNPS	10.3390/ijms232315054
11	Aloesin	Chromone	GNPS	10.1186/s12906-015-0803-4
12	Myricetin	Flavonol	GNPS	10.3390/molecules29102354
13	Luteolin	Flavone	GNPS &SIRIUS	10.3390/ijms17111947
14	Manool	Labdane diterpenoid	GNPS	10.1021/acs.jnatprod.9b01024
15	Apigenin	Flavone	GNPS	10.3390/molecules26071980
16	Diosmetin	Flavone	GNPS	10.1155/2022/9966750
17	Tricetin	Flavone	GNPS &SIRIUS	10.1016/j.micpath.2019.103928
18	Puerarin	Isoflavone	GNPS & SIRIUS	10.1186/s12879-025-10951-1
19	Rhamnetin	Flavonol	GNPS	10.3390/ijms232112895
20	Lariciresinol	Furanoid lignan	GNPS	10.1128/spectrum.00693-25
21	Myricitrin	Flavonol	GNPS	10.1186/s12906-020-03139-4
22	Alpinetin	Flavanone	GNPS	10.3389/fphar.2022.814370
23	Isoliquiritigenin	Chalcone	GNPS	10.1186/s12866-025-04298-5
24	Galangin	Flavonol	GNPS	10.1016/j.phymed.2004.07.003
25	Isopimaric acid	Isopimarane diterpenoid	GNPS	10.1002/ptr.1711
26	Chlorogenic Acid	Phenolic acid	SIRIUS	10.1155/2013/392058
27	Isobiflorin	Phenolic glycoside	SIRIUS	10.22377/ajp.v13i3.3292
28	Biflorin	Phenolic glycoside	SIRIUS	10.1016/j.bmcl.2015.11.095
