# Supplementary material for: Integrative LC-HR-QTOF-MS and Computational Metabolomics Approaches for Compound Annotation, Chemometric Profiling and In Silico Antibacterial Evaluation of Ugandan Propolis
Source: Metabolites. 2026 Feb 3;16(2):109. doi: 10.3390/metabo16020109 (PMC12942557; doi:10.3390/metabo16020109)
Supplement: Supplementary file 1 [file metabolites-16-00109-s001.zip › Supplementary Table S5-In silico ADME characteristics and medicinal chemistry descriptors for ligands.pdf]

Supplementary Table S5. *In silico* ADME characteristics and medicinal chemistry descriptors for ligands L2–L9, including predicted absorption, distribution, metabolism, excretion, and drug-likeness parameters

ADME phase		Ligands						
		L2	L3	L4	L6	L7	L8	L9
Absorption	Caco-2 Permeability	-6.134	-5.853	-6.593	-4.754	-5.09	-5.11	-4.795
	MDCK Permeability	0	0	0	0	0	0	0
	Pgp inhibitor	No	No	No	Yes	No	No	Yes
	F20%	>20%	<20%	>20%	>20%	<20%	<20%	>20%
Distribution	BBB	No	No	No	No	No	No	No
	PPB	83.60%	83.60%	84.70%	97.40%	70.20%	94.50%	84.90%
	Fraction unbound in plasma	14.80%	18.20%	13.30%	2.60%	30.20%	7.50%	13.70%
Metabolism	CYP1A2 inhibitor	No	No	No	No	No	No	No
	HLM	Yes	Yes	Yes	Yes	Yes	Yes	Yes
	CYP3A4 inhibitor	No	No	No	No	No	Yes	No
	CYP2C19 inhibitor	No	No	No	No	No	Yes	Yes
Excretion	Plasma Clearance	3.685	4.038	2.006	2.654	2.884	6.745	8.054
	Half Life	3.212	1.83	3.677	1.121	1.57	1.523	2.912
Medicinal Chemistry	QED	0.277	0.323	0.223	0.711	0.180	0.773	0.753
	Lipinski Rule	Rejected	Accepted	Rejected	Accepted	Accepted	Accepted	Accepted
	Pfizer Rule	Accepted	Accepted	Accepted	Rejected	Accepted	Accepted	Accepted
	GSK Rule	Rejected	Rejected	Rejected	Rejected	Rejected	Accepted	Accepted
	GoldenTriangle	Accepted	Accepted	Accepted	Accepted	Rejected	Accepted	Accepted
